# Supplementary material for: Polygenic signals of sex differences in selection in humans from the UK Biobank
Source: PLoS Biol. 2022 Sep 6;20(9):e3001768. doi: 10.1371/journal.pbio.3001768 (PMC9481184; doi:10.1371/journal.pbio.3001768)
Supplement: S1 Appendix — Section A. Theoretical null distributions for FST estimates. Section B. Hitchhiking estimates in between-sex FST. Section C. Defining upper bounds for excess heterozygosity in FIS estimates arising from SA selection. Section D. Polygenicity of signals of sex differences in selection. Section E. Null model for unfolded reproductive FST. Section F. Correcting for sex-specific population structure. Section G. Sex differences in selection and the relation between FST and MAF. Section H. Associations between metrics of sex differences in selection and MAF. Section I. Associations between metrics of sex differences in selection and candidates for balancing selection. (PDF) [file pbio.3001768.s001.pdf]

SI Appendix, Section A: Theoretical null distributions for F_{ST} estimates

In the following sections, we outline null models for between-sex F_{ST} metrics that potentially capture sex-differential effects of genetic variation on pre-adult viability ("adult F_{ST}"), on adult reproductive success ("reproductive F_{ST}"), and on total fitness ("gametic F_{ST}"). In each case, we follow bi-allelic loci, each with alleles labelled A_1 and A_2, and their frequencies in adults of each sex or the gametes contributing to production of offspring.

Adult F_{ST}

In the absence of sex differences in viability selection, the frequencies of autosomal alleles, which are equalized at fertilization, remain equal between adults of each sex. Random sampling of individuals included within a panel of sequenced adults will, nevertheless, generate non-zero estimates of between-sex F_{ST} (*i.e.*, non-zero adult $\hat{F}_{ST}$). These sex differences arise from error in estimating female and male allele frequencies at each locus.

Under a null model in which there are no sex differences in viability selection, and the population is at Hardy-Weinberg equilibrium for the locus, Ruzicka et al. [1] showed that, $2n_H\hat{F}_{ST}$ follows a chi-squared distribution with 1 degree of freedom, where $n_H = 2\left(1/n_f + 1/n_m\right)^{-1}$ is the harmonic mean sample size of female- and male-derived sequences for the locus (*i.e.*, $n_f = 2N_f$ and $n_m = 2N_m$, where N_f and N_m are the female and male sample sizes, and the "2" accounts for diploidy) (see their Appendix A). This theoretical distribution for adult $\hat{F}_{ST}$ under the null (along with those developed below), applies well for large datasets (large n_H) in which very rare polymorphic loci are excluded prior to analysis.

Their result can be generalized to cases where the population deviates from Hardy-Weinberg equilibrium, in which case:

$$\hat{F}_{ST} \approx \frac{(1 - F_{IS})}{4}\left(\frac{1}{2N_f} + \frac{1}{2N_f}\right)X = \frac{(1 - F_{IS})}{2n_H}X$$

where X is a chi-squared random variable with 1 degree of freedom, and $F_{IS} = \frac{p_{12}}{2p(1-p)} - 1$ is the deviation of the population from Hardy-Weinberg equilibrium (HWE). We follow Kasimatis et al. (2019) in the manner with which we define F_{IS} with positive values ($F_{IS} > 0$) corresponding to an excess of heterozygotes relative to HWE, and negative values ($F_{IS} < 0$) corresponding to a deficiency of heterozygotes.

Gametic F_{ST}

Let M_{ij} be the total number of offspring produced by males with genotype ij ($ij = {}_{11}$ for A_1A_1 individuals, $ij = {}_{12}$ for A_1A_2 and $ij = {}_{22}$ for A_2A_2). The frequency of the A_1 allele in male gametes contributing to offspring will be:

$$\frac{M_{11} + x_{12}}{M_{11} + M_{12} + M_{22}}$$

where x_{12} is a binomially distributed random variable with mean and variance of $E(x_{12}) = M_{12}/2$ and $\text{var}(x_{12}) = M_{12}/4$. Thus, the expected frequency of the A_1 allele in gametes transmitted by males to their offspring is:

$$\hat{p}'_m = \frac{M_{11} + \frac{1}{2}M_{12}}{M_{11} + M_{12} + M_{22}}$$

Similarly, letting F_{ij} represent the total number of offspring produced by females with genotype ij, the expected frequency of A_1 in female gametes contributing to offspring is:

$$\hat{p}'_f = \frac{F_{11} + \frac{1}{2}F_{12}}{F_{11} + F_{12} + F_{22}}$$

In the absence of selection, there will be two sources of variability affecting the values of M_{ij} and F_{ij}. First, there will be random variability in the numbers of individuals of each genotype within the sample of adults. For example, in a random sample of N_m males, the number of individuals of with genotypes 11, 12, and 22 (*i.e.*, A_1A_1, A_1A_2, A_2A_2), denoted by the vector $\boldsymbol{n} = n_{11}, n_{12}, n_{22}$, will follow a multinomial distribution with parameters N_m, p_{11}, p_{12}, and p_{22}, where p_{ij} represents the frequency of genotype ij (note that the frequency of the A_1 allele is $p = p_{11} + p_{12}/2$ and the frequency of the A_2 allele is $1 - p = p_{22} + p_{12}/2$). Second, there will be random variability in the number of offspring produced by each individual in the population. For the case where the genotype has no effect on reproductive success, then the offspring number, per male, follows a distribution with a mean and variance of μ_m and σ_m^2 that is independent of genotype. Likewise, the offspring number, per female, follows a distribution with a mean and variance of μ_f and σ_f^2 that is independent of genotype. Values of μ_f, σ_f^2, μ_m and σ_m^2 can be estimated from the females and males represented in the UK Biobank dataset.

The expectation of $\hat{p}'_m$, conditioned on the numbers of individuals per genotype, is:

$$\mathrm{E}[\hat{p}'_m|\boldsymbol{n}] = \mathrm{E}\left[\frac{M_{11}+\frac{1}{2}M_{12}}{M_{11}+M_{12}+M_{22}}\middle|\boldsymbol{n}\right]$$

$$\approx \frac{\mathrm{E}\left[M_{11}+\frac{1}{2}M_{12}\middle|\boldsymbol{n}\right]}{\mathrm{E}[M_{11}+M_{12}+M_{22}|\boldsymbol{n}]} - \frac{\mathrm{cov}\left(M_{11}+\frac{1}{2}M_{12}, M_{11}+M_{12}+M_{22}\middle|\boldsymbol{n}\right)}{\mathrm{E}[M_{11}+M_{12}+M_{22}|\boldsymbol{n}]^2}$$

$$+\frac{\mathrm{E}\left[M_{11}+\frac{1}{2}M_{12}\middle|\boldsymbol{n}\right]\mathrm{var}(M_{11}+M_{12}+M_{22}|\boldsymbol{n})}{\mathrm{E}[M_{11}+M_{12}+M_{22}|\boldsymbol{n}]^3} = \hat{p}$$

where $\hat{p} = \left(n_{11}+\frac{1}{2}n_{12}\right)N_m^{-1}$ and $\hat{p}_{ij} = n_{ij}N_m^{-1}$. The approximation is based on a Taylor series expansion of $\hat{p}'_m$. The conditional means and variances for the M_{ij} are:

$$\mathrm{E}\left[M_{ij}\middle|\boldsymbol{n}\right] = \mathrm{E}\left[\sum_{k=1}^{n_{ij}} m_k\right] = n_{ij}\mu_m$$

$$\mathrm{var}\left[M_{ij}\middle|\boldsymbol{n}\right] = \mathrm{var}\left[\sum_{k=1}^{n_{ij}} m_k\right] = \sum_{k=1}^{n_{ij}} \mathrm{var}(m_k) = n_{ij}\sigma_m^2$$

where the m_k are IID random variables with mean and variance of μ_m and σ_m^2, corresponding to the mean and variance for the numbers of offspring reported by males from the population. From the law of total expectation, the expected value of $\hat{p}'_m$ becomes:

$$\mathrm{E}[\hat{p}'_m] \approx \mathrm{E}[\hat{p}] = p$$

The variance for $\hat{p}'_m$, conditioned on the numbers of individuals per genotype, is:

$$\mathrm{var}[\hat{p}'_m|\boldsymbol{n}] = \mathrm{var}\left[\frac{M_{11}+\frac{1}{2}M_{12}}{M_{11}+M_{12}+M_{22}}\middle|\boldsymbol{n}\right]$$

$$\approx \frac{\mathrm{var}\left[M_{11}+\frac{1}{2}M_{12}\middle|\boldsymbol{n}\right]}{\mathrm{E}[M_{11}+M_{12}+M_{22}|\boldsymbol{n}]^2}$$

$$-2E\left[M_{11}+\frac{1}{2}M_{12}\middle|\boldsymbol{n}\right]\frac{\mathrm{cov}\left(M_{11}+\frac{1}{2}M_{12}, M_{11}+M_{12}+M_{22}\middle|\boldsymbol{n}\right)}{\mathrm{E}[M_{11}+M_{12}+M_{22}|\boldsymbol{n}]^3}$$

$$+\frac{\mathrm{E}\left[M_{11}+\frac{1}{2}M_{12}\middle|\boldsymbol{n}\right]^2}{\mathrm{E}[M_{11}+M_{12}+M_{22}|\boldsymbol{n}]^4}\mathrm{var}(M_{11}+M_{12}+M_{22}|\boldsymbol{n})$$

$$= \frac{1}{N_m}\frac{\sigma_m^2}{\mu_m^2}\left[\frac{n_{11}+\frac{1}{4}n_{12}}{N_m} - \left(\frac{n_{11}+\frac{1}{2}n_{12}}{N_m}\right)^2\right]$$

From the law of total variance, we have:

$$\text{var}[\hat{p}'_m] \approx \frac{1}{N_m}\frac{\sigma_m^2}{\mu_m^2}\left(p(1-p) - \frac{1}{4}p_{12}\right) + \frac{p_{11}(1-p_{11}) + \frac{1}{4}p_{12}(1-p_{12}) - p_{11}p_{12}}{N_m} + O(N_m^{-2})$$

Ignoring terms of $O(N_m^{-2})$, we obtain:

$$\text{var}[\hat{p}'_m] \approx \frac{1}{N_m}\left(1 + \frac{\sigma_m^2}{\mu_m^2}\right)\left(p(1-p) - \frac{1}{4}p_{12}\right) = \frac{p(1-p)}{2N_m}\left(1 + \frac{\sigma_m^2}{\mu_m^2}\right)(1 - F_{IS})$$

where $F_{IS} = \frac{p_{12}}{2p(1-p)} - 1$ represents the population's deviation from Hardy-Weinberg equilibrium for the locus. If the population is at HWE, then we have:

$$E[\hat{p}'_m] \approx p$$

$$\text{var}[\hat{p}'_m] \approx \frac{p(1-p)}{2N_m}\left(1 + \frac{\sigma_m^2}{\mu_m^2}\right)$$

Equivalent expressions for females are obtained by replacing "m" subscripts with "f".

With large sample sizes, the difference between the projected allele frequencies in the gametes of each sex will be approximately normally distributed:

$$\hat{p}'_f - \hat{p}'_m \sim N\left(0, \frac{p(1-p)}{2}\left[\frac{1}{N_f}\left(1 + \frac{\sigma_f^2}{\mu_f^2}\right) + \frac{1}{N_m}\left(1 + \frac{\sigma_m^2}{\mu_m^2}\right)\right](1 - F_{IS})\right)$$

Consequently, the projected gametic F_{ST} for the sample will be:

$$\hat{F}_{ST} \approx \frac{\left(\hat{p}'_f - \hat{p}'_m\right)^2}{4p(1-p)} \approx (1 - F_{IS})\left[\frac{1}{8N_f}\left(1 + \frac{\sigma_f^2}{\mu_f^2}\right) + \frac{1}{8N_m}\left(1 + \frac{\sigma_m^2}{\mu_m^2}\right)\right]X$$

where X is a chi-squared random variable with 1 degree of freedom. If the population is at HWE, then we have:

$$\hat{F}_{ST} \approx \frac{\left(\hat{p}'_f - \hat{p}'_m\right)^2}{4p(1-p)} \approx \left[\frac{1}{8N_f}\left(1 + \frac{\sigma_f^2}{\mu_f^2}\right) + \frac{1}{8N_m}\left(1 + \frac{\sigma_m^2}{\mu_m^2}\right)\right]X$$

Reproductive F_{ST}

Adult F_{ST} potentially captures effects of sex differences in viability selection, whereas gametic F_{ST} potentially captures sex differences in selection through any fitness component. To isolate the effect of sex differences in selection through components of adult reproductive success, we require a measure of allele frequency divergence between the sexes that reflects the variation in reproductive success among reproductively mature adults, and which does not include (or removes the effect of) allele frequency differences between sexes in the adult samples. Specifically, we wish to test for between-sex divergence in projected gametic allele frequencies

(*i.e.*, differences between $\hat{p}'_f$ and $\hat{p}'_m$) beyond what can be explained by allele frequency differences between females and males within the sample of adults.

Let $\hat{p}_f$ and $\hat{p}_m$ represent the female and male allele frequencies estimated from the adult samples, and $\hat{p} = \frac{1}{2}(\hat{p}_f + \hat{p}_m)$ represent their average. A measure of the amount of allele frequency divergence arising from differential reproduction between the sexes is given by:

$$\delta = (\hat{p}'_f - \hat{p}_f) - (\hat{p}'_m - \hat{p}_m) = (\hat{p}'_f - \hat{p}'_m) - (\hat{p}_f - \hat{p}_m)$$

where $\hat{p}'_f$ and $\hat{p}'_m$ are the projected gametic allele frequencies. From this expression, we will establish a null model for the projected gametic allele frequencies of each sex given the estimated allele frequencies in the adults. In our null model (outlined below), we will assume there are no intrinsic differences in reproductive success associated with each genotype or sex. This null model is similar to the gametic F_{ST} null model (above) in that it accounts for random variation in reproductive success. It differs from the gametic F_{ST} model by discounting random sampling effects on sex-specific allele frequency estimates from adults (*i.e.*, $\hat{p}_f$ and $\hat{p}_m$ are treated as constants in what follows).

For very large adult samples (large N_f and N_m, as in the UK Biobank) the null distributions for $\hat{p}'_f$ and $\hat{p}'_m$ (each conditioned on the allele frequencies in adults, $\hat{p}_f$ and $\hat{p}_m$) will each be approximately normal with mean and variance for the jth sex given by:

$$\mathrm{E}[\hat{p}'_j] = \hat{p}_j$$

$$\mathrm{var}[\hat{p}'_j] = \frac{1}{N_j}\frac{\sigma_j^2}{\mu_j^2}\left[\hat{p}_j(1-\hat{p}_j) - \frac{1}{4}\hat{p}_{12,j}\right] = \frac{\hat{p}_j(1-\hat{p}_j)}{2N_j}\frac{\sigma_j^2}{\mu_j^2}(1-\hat{F}_{IS,j})$$

where μ_j and σ_j^2 refer to the mean and variance for reproductive success of the jth sex (as defined for gametic F_{ST}), and $\hat{F}_{IS,j} = \frac{\hat{p}_{12,j}}{2\hat{p}_j(1-\hat{p}_j)} - 1$ is the deviation of the sample of genotypes from Hardy-Weinberg equilibrium (HWE). Recall that $\hat{F}_{IS,j} > 0$ corresponds to an excess of heterozygotes in our model; $\hat{F}_{IS,j} < 0$ corresponds to a deficiency of heterozygotes. The approach to these results parallels the derivation for gametic F_{ST} null model. When there is no deviation from HWE ($\hat{F}_{IS,j} = 0$), the variance further simplifies to

$$\mathrm{var}[\hat{p}'_j] = \frac{\hat{p}_j(1-\hat{p}_j)}{2N_j}\frac{\sigma_j^2}{\mu_j^2}$$

The null distribution for δ will, therefore, be approximately normal with mean and variance:

$$\mathrm{E}[\delta] = \mathrm{E}[(\hat{p}'_f - \hat{p}'_m) - (\hat{p}_f - \hat{p}_m)] = 0$$

$$\mathrm{var}[\delta] = \mathrm{var}\left[\left(\hat{p}'_f - \hat{p}'_m\right) - \left(\hat{p}_f - \hat{p}_m\right)\right]$$

$$\approx \frac{\hat{p}_f\left(1-\hat{p}_f\right)}{2N_f}\frac{\sigma_f^2}{\mu_f^2}\left(1-\hat{F}_{IS,f}\right) + \frac{\hat{p}_m(1-\hat{p}_m)}{2N_m}\frac{\sigma_m^2}{\mu_m^2}\left(1-\hat{F}_{IS,m}\right)$$

Let us define the reproductive F_{ST} statistic as:

$$\hat{F}_{ST} = \frac{\delta^2}{4\hat{p}(1-\hat{p})}$$

Under our null model, the estimate of reproductive F_{ST} for a locus will be:

$$\hat{F}_{ST} \approx \frac{\frac{\hat{p}_f\left(1-\hat{p}_f\right)}{2N_f}\frac{\sigma_f^2}{\mu_f^2}\left(1-\hat{F}_{IS,f}\right) + \frac{\hat{p}_m(1-\hat{p}_m)}{2N_m}\frac{\sigma_m^2}{\mu_m^2}\left(1-\hat{F}_{IS,m}\right)}{4\hat{p}(1-\hat{p})}X$$

where X is a chi-squared random variable with one degree of freedom. The result follows from the fact that the distribution of $\delta/\sqrt{\mathrm{var}[\delta]}$ has standard normal distribution (approximately). In the special case where female and male allele frequencies in the sample are approximately equal (as is the case for the UK Biobank sites that pass quality control in our analysis), the null model for reproductive F_{ST} will further simplify to:

$$\hat{F}_{ST} \approx \left(\frac{1}{8N_f}\frac{\sigma_f^2}{\mu_f^2}\left(1-\hat{F}_{IS,f}\right) + \frac{1}{8N_m}\frac{\sigma_m^2}{\mu_m^2}\left(1-\hat{F}_{IS,m}\right)\right)X$$

SI Appendix, Section B: Hitchhiking effects in between-sex F_{ST}

For a causal locus that differentially affects female and male fitness, the expected inflation of between-sex F_{ST} is given by:

$$F_{ST} \approx \frac{pq}{16}\left(\frac{d\ln(\bar{w}_f)}{dp} - \frac{d\ln(\bar{w}_m)}{dp}\right)^2$$

in which the derivatives capture the effect of the causal locus on female and male fitness (see *SI Appendix*, Section G).

Polymorphic loci that are physically linked to a given causal locus, and in linkage disequilibrium (LD) with it, will also exhibit inflated F_{ST}, on average. Let x refer to the frequency of one of a pair of alleles at a neutral locus that is linked to a selected locus. The expected within generation change in frequency of the neutral allele will be:

$$\Delta x_f \approx \frac{D}{2} \cdot \frac{d\ln(\bar{w}_f)}{dp}$$

in females, and:

$$\Delta x_m \approx \frac{D}{2} \cdot \frac{d\ln(\bar{w}_m)}{dp}$$

in males of the population, where D is the degree of linkage disequilibrium between the neutral and the causal locus. The expected inflation of between-sex F_{ST} at the neutral site is given by:

$$F_{ST} \approx \frac{D^2}{16x(1-x)}\left(\frac{d\ln(\bar{w}_f)}{dp} - \frac{d\ln(\bar{w}_m)}{dp}\right)^2 = \rho^2 \frac{pq}{16}\left(\frac{d\ln(\bar{w}_f)}{dp} - \frac{d\ln(\bar{w}_m)}{dp}\right)^2$$

where $\rho^2 = D^2(x(1-x)pq)^{-1}$ is the squared correlation coefficient between the neutral locus and the causal locus. From the final result, we see that each neutral locus in LD with the causal site will hitchhike along with it, leading to an inflation of F_{ST} at hitchhiking loci that is proportional to the F_{ST} at the causal locus and the square of the correlation coefficient between hitchhiking and causal loci.

SI Appendix, Section C: Defining upper bounds for excess heterozygosity in F_{IS} estimates arising from SA selection

Deviations from Hardy-Weinberg equilibrium (HWE) potentially reflect artefacts that we wish to eliminate from our analysis. However, SA selection is predicted to generate excess heterozygosity relative to predictions under Hardy-Weinberg equilibrium. We only wish to remove loci with deviations from HWE that are too pronounced to be explained by SA selection. To define the plausible range of HWE deviations under SA selection, we use the $\hat{F}_{IS}$ statistic to define the estimated deviation:

$$\hat{F}_{IS} = \frac{P_{Aa}}{2\bar{p}(1-\bar{p})} - 1$$

where P_{Aa} is the frequency of heterozygotes at the locus, and $\bar{p}$ is the sex-averaged allele frequency. For a locus under SA selection, let p_f and p_m represent the frequency of the female-beneficial allele in eggs and sperm contributing to fertilization in a given generation (respectively). In a random sample of n individuals from the offspring cohort, $\hat{F}_{IS}$ for the locus will be a random variable from a normal distribution with mean and variance of:

$$\mathrm{E}[\hat{F}_{IS}] = \frac{1}{2n} + \frac{(p_f - p_m)^2}{4\bar{p}(1-\bar{p})}$$

$$\mathrm{var}[\hat{F}_{IS}] = \frac{1}{n}$$

[1]. Thus, we expect some degree of deviation from HWE owing to sex differences in selection.

For a SA locus at polymorphic equilibrium and additive fitness effects in each sex, the equilibrium allele frequency difference between sexes after selection is:

$$p_f - p_m = \frac{2(1-ps_m)}{s_m}\left(\sqrt{1 + \frac{s_f s_m p(1-p)}{(1-ps_m)(1-(1-p)s_f)}} - 1\right)$$

where $p = \bar{p} = (s_f - s_m + s_f s_m)/2s_f s_m$ at equilibrium [2]. If we let p represent the minor allele frequency, then at equilibrium, we have:

$$\frac{(p_f - p_m)}{4\bar{p}(1-\bar{p})} = \frac{1}{4p(1-p)}\left(\frac{2(1-ps_{\max})}{s_{\max}}\sqrt{1 + p(1-p)\left(\frac{s_{\max}}{1-ps_{\max}}\right)^2} - \frac{2(1-ps_{\max})}{s_{\max}}\right)^2$$

where $s_{\max} = \max(s_m, s_f)$. With sufficiently small $s_{\max}$, the last expression can be approximated as:

$$\frac{(p_f - p_m)}{4\bar{p}(1-\bar{p})} = \frac{p(1-p)}{4}\left(\frac{s_{\max}}{1-ps_{\max}}\right)^2$$

which gives us:

$$\mathrm{E}\big[\hat{F}_{IS}\big] \approx \frac{1}{2n} + \frac{p(1-p)}{4}\left(\frac{s_{\max}}{1-ps_{\max}}\right)^2$$

$$\mathrm{var}\big[\hat{F}_{IS}\big] = \frac{1}{n}$$

The approximation is accurate for $s_{\max}$ = 0.2. The following plot shows the exact and approximate results for n = 250,000 and $s_{\max}$ = 0.2.

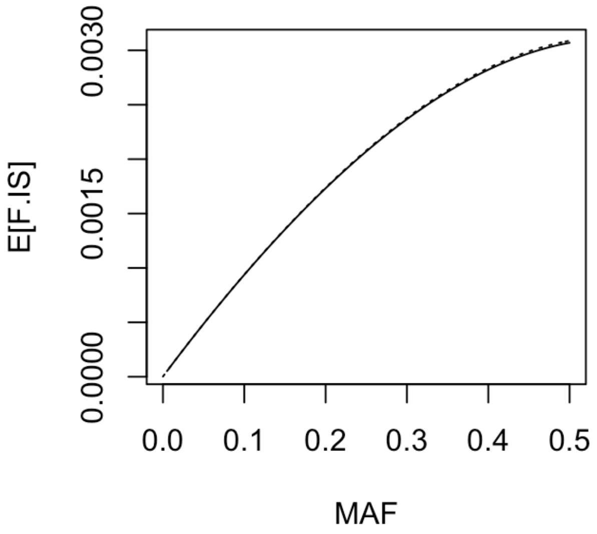

SI Appendix, Section D: Polygenicity of signals of sex differences in selection

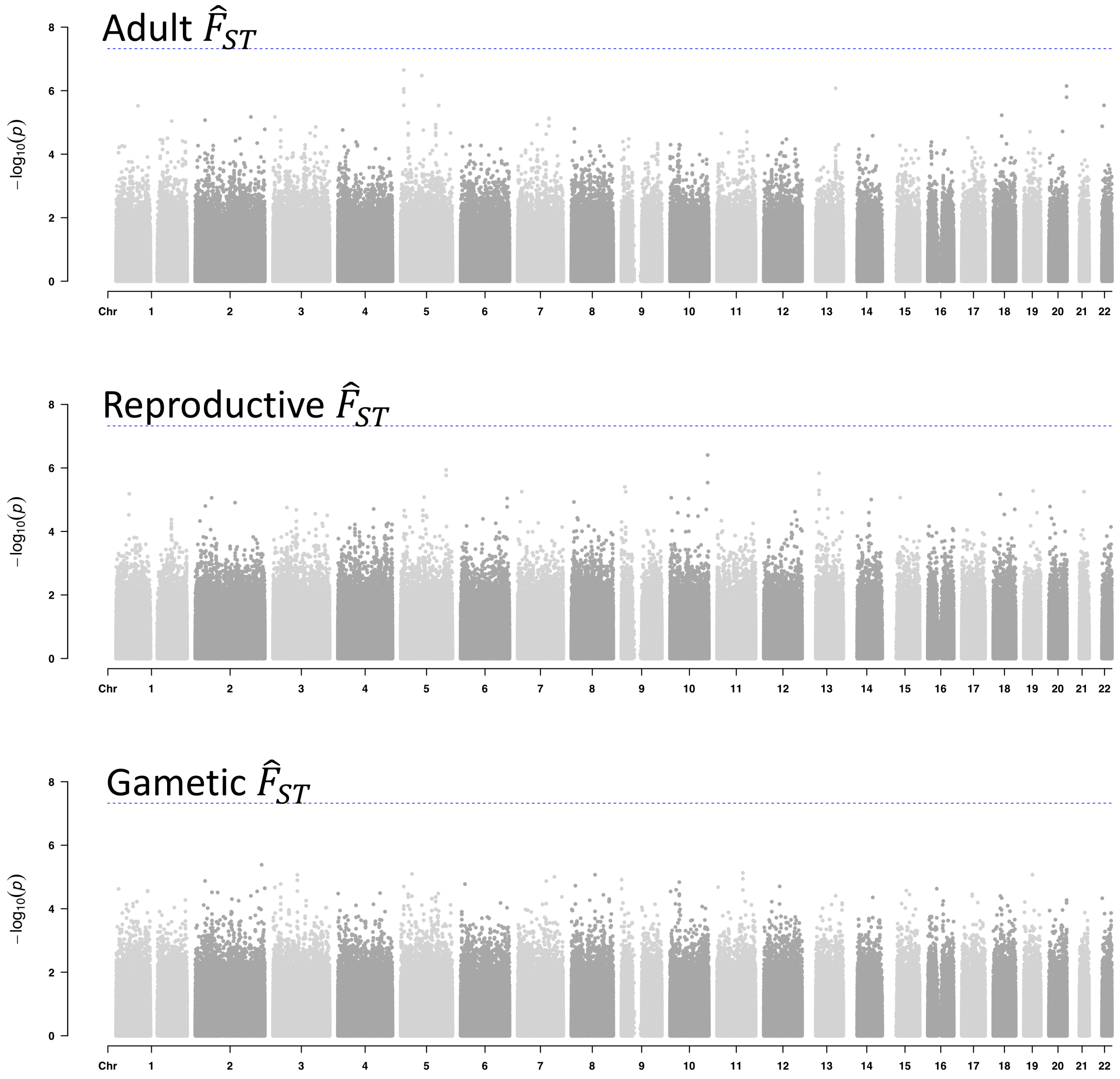

Fig. SD1. Manhattan plots for F_{ST} metrics of sex differences in selection. P-values were obtained by specifying observed values of $\hat{F}_{ST}$ (scaled by the relevant null for each locus, such that the overall distribution across loci is chi-square under the null, as per Materials and Methods) as quantiles in the cumulative distribution function of a chi-square. Blue dashed line represents the Bonferroni-corrected p-value threshold. The code and data needed to generate this Figure can be found at https://github.com/filipluca/polygenic_SA_selection_in_the_UK_biobank and https://zenodo.org/record/6824671

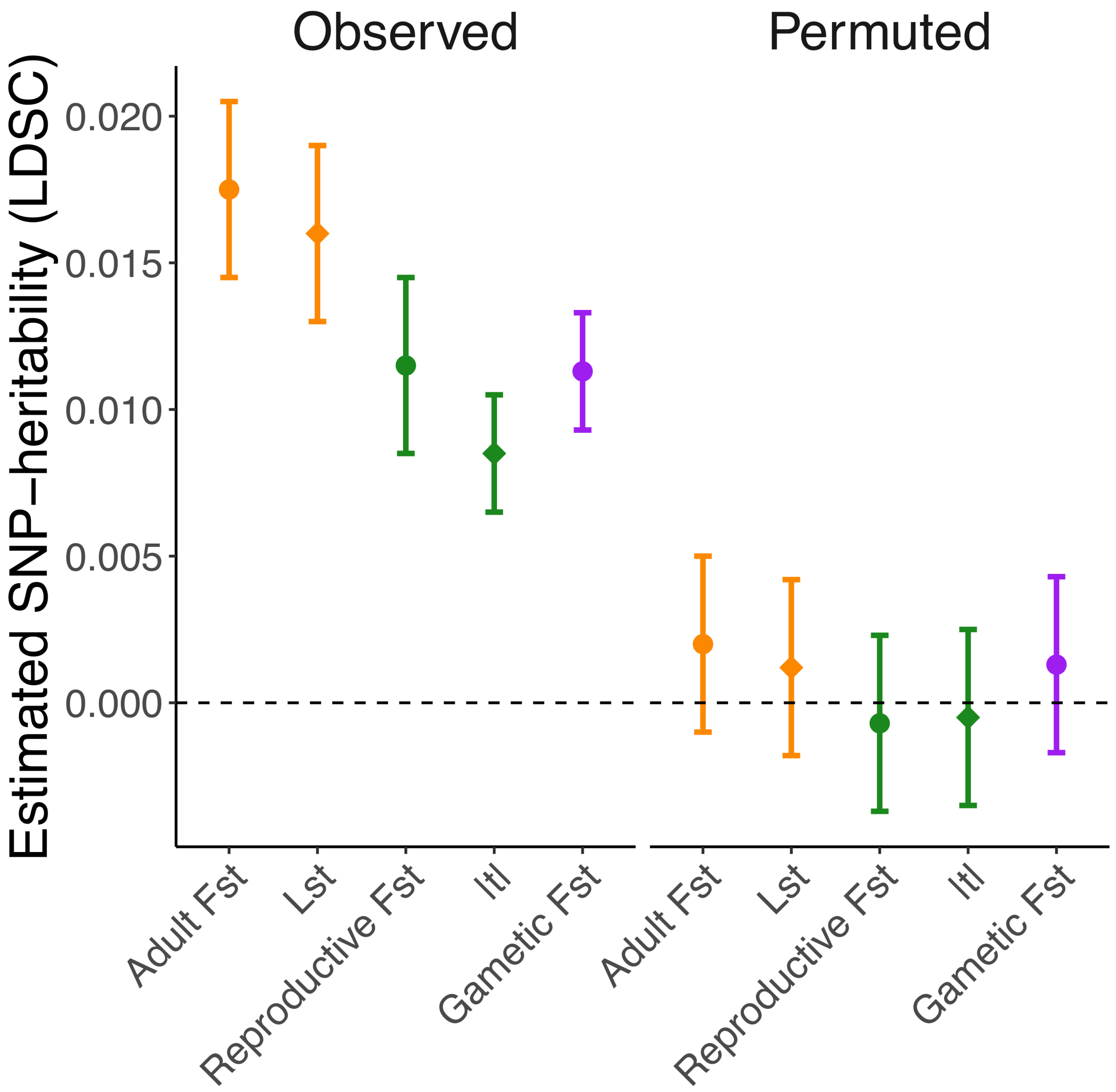

Fig. SD2. SNP-heritability of each metric of sex-differential selection. Estimates of SNP-heritability for each metric were estimated using Stratified LDscore regression, implementing the "full baseline model" in Finucane et al. [3]. The model accounts for potentially non-random contributions of different functional categories to overall SNP-heritability. The code and data needed to generate this Figure can be found at https://github.com/filipluca/polygenic_SA_selection_in_the_UK_biobank and https://zenodo.org/record/6824671

SI Appendix, Section E: Null Model for unfolded Reproductive F_{ST}

The elevation of reproductive F_{ST} relative to our null model is a genome-wide signal of sex-differential selection, though in principle, the signal may have arisen because of sex-differences in the strength of selection (sexually concordant or SC selection), due to loci with sex-limited effects (SL selection), or because of sex differences in the direction of selection (SA selection).

These three mechanisms can be distinguished as follows. First, consider the divergence of the projected allele frequency in males ($\hat{p}'_m$) relative to the observed frequency ($\hat{p}_m$). Under the null, the genotypes of the locus have no effect on male reproductive success, and therefore:

$$\mathrm{E}(\hat{p}'_m - \hat{p}_m) = 0$$

$$\mathrm{var}(\hat{p}'_m - \hat{p}_m) = \mathrm{var}(\hat{p}'_m) = \frac{\hat{p}_m(1-\hat{p}_m)}{2n_m}\frac{\sigma_m^2}{\mu_m^2}\left(1-\hat{F}_{IS}^m\right)$$

Under the null, the following standardized metric will follow a standard normal distribution:

$$x = \frac{(\hat{p}'_m - \hat{p}_m)}{\sqrt{\frac{\hat{p}_m(1-\hat{p}_m)}{2n_m}\frac{\sigma_m^2}{\mu_m^2}\left(1-\hat{F}_{IS}^m\right)}}$$

The same applies to females:

$$y = \frac{(\hat{p}'_f - \hat{p}_f)}{\sqrt{\frac{\hat{p}_f(1-\hat{p}_f)}{2n_f}\frac{\sigma_f^2}{\mu_f^2}\left(1-\hat{F}_{IS}^f\right)}}$$

Under SC selection, we expect $(\hat{p}'_m - \hat{p}_m)(\hat{p}'_f - \hat{p}_f) > 0$, on average. Under SL selection, we expect $(\hat{p}'_m - \hat{p}_m)(\hat{p}'_f - \hat{p}_f) = 0$. And under SA selection, we expect $(\hat{p}'_m - \hat{p}_m)(\hat{p}'_f - \hat{p}_f) < 0$, on average. We know, from above, that under the null model, x and y are independent and follow standard normal distributions. Moreover, their product, $z = xy$, should be symmetric with a mean of zero, a variance of one, and well-defined tails as outlined above. The following metric, which we term "unfolded reproductive F_{ST}", can be compared to that null distribution:

$$\frac{(\hat{p}'_m - \hat{p}_m)(\hat{p}'_f - \hat{p}_f)}{\sqrt{\frac{\hat{p}_m(1-\hat{p}_m)}{2n_m}\frac{\sigma_m^2}{\mu_m^2}\left(1-\hat{F}_{IS}^m\right)\frac{\hat{p}_f(1-\hat{p}_f)}{2n_f}\frac{\sigma_f^2}{\mu_f^2}\left(1-\hat{F}_{IS}^f\right)}}$$

SA selection should lead to inflation in the lower quantiles of the distribution and SC selection should lead to inflation in the upper quantiles. If we mostly see inflation in the lower quantiles, then SA selection would appear to be the dominant factor in the inflation of reproductive F_{ST}. If it is primarily the upper quantiles that are inflated, then SC selection predominates. If we see symmetric inflation, this could imply a mixture of SA and SC loci contributing to the inflation of reproductive F_{ST}.

SI Appendix, Section F: Correcting for sex-specific population structure

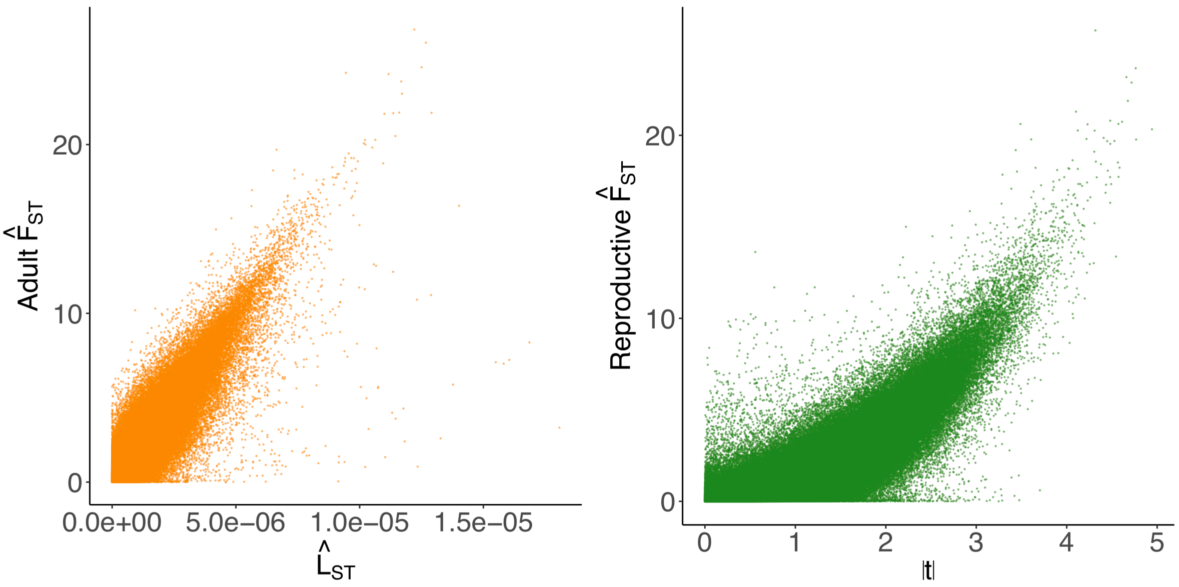

Fig. SF1. Scatter plots between values of complementary metrics of sex-differential selection. $\hat{F}_{ST}$ values have been scaled by the multiplier of the relevant null for each locus (such that the overall distribution across loci is chi-square with one degree of freedom under the null, as per Materials and Methods). The code and data needed to generate this Figure can be found at https://github.com/filipluca/polygenic_SA_selection_in_the_UK_biobank and https://zenodo.org/record/6824671

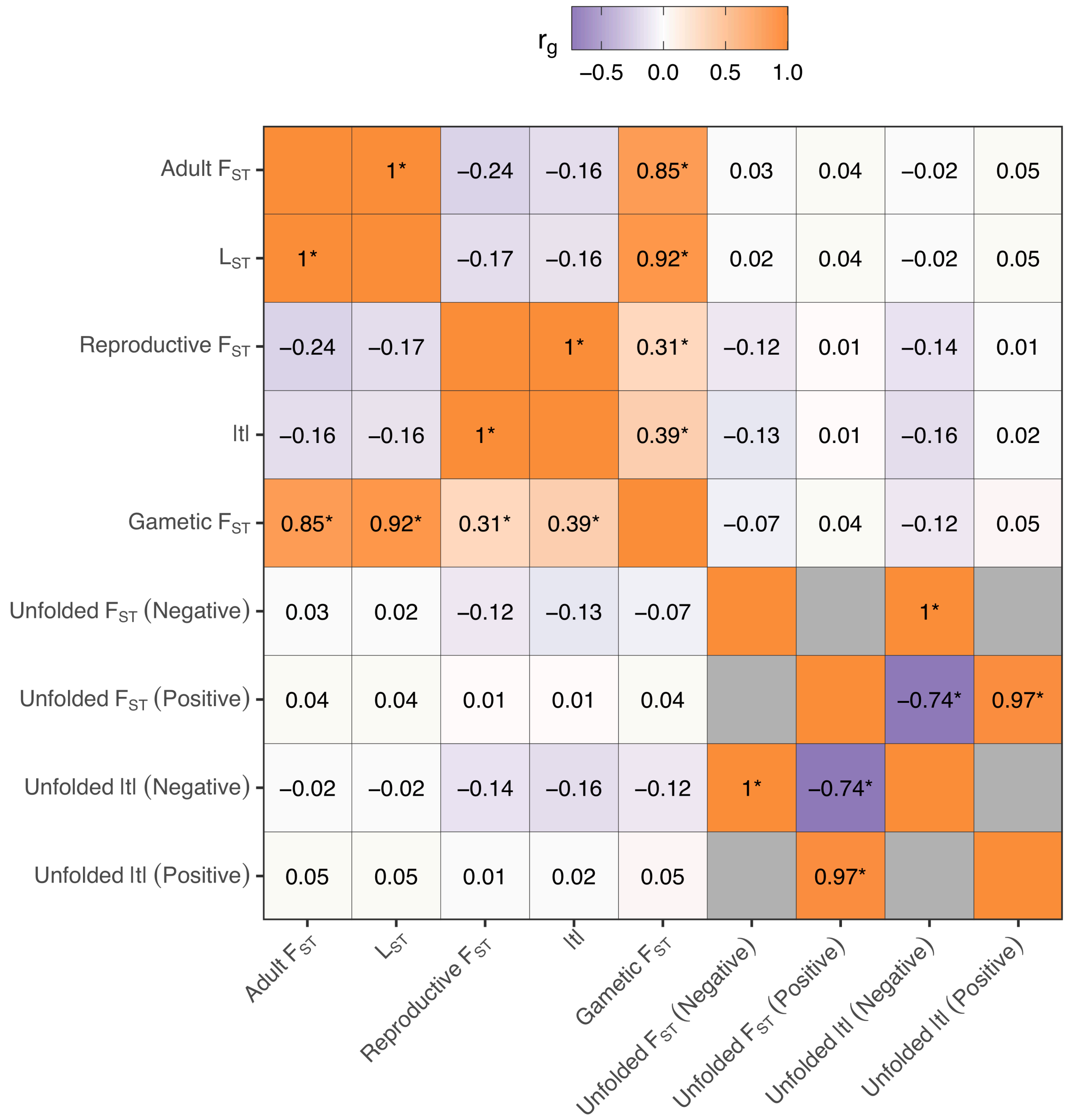

Fig. SF2. Genetic correlations between metrics of sex-differential selection. Same as Fig. 5A, but presented for all metrics of sex-differential selection. There are no sites with both positive and negative values of unfolded reproductive $\hat{F}_{ST}$ (or unfolded t), hence absent genetic correlations for those combinations. The code and data needed to generate this Figure can be found at The code and data needed to generate this Figure can be found at https://github.com/lukeholman/UKBB_LDSC and https://zenodo.org/record/6824671

SI Appendix, Section G: Sex differences in selection and the relation between F_{ST} and MAF

The following calculations are based on models of sex differences in viability selection, though they apply qualitatively to selection through other fitness components. We consider a population of adults in which the true allele frequencies are p_f and p_m (after viability selection), and p is the frequency at fertilization. As shown in Ruzicka et al. [1], the expected value of the estimate of F_{ST} is approximately:

$$\mathrm{E}[\hat{F}_{ST}] = \frac{n_m p_f(1-p_f) + n_f p_m(1-p_m)}{4n_f n_m p(1-p)} + \frac{(p_f - p_m)^2}{4p(1-p)}$$

$$= \frac{1}{2n_H} + \frac{(n_m - n_f)(1-2p)(p_f - p_m)}{8n_f n_m p(1-p)} + \left(1 - \frac{1}{2n_H}\right)\frac{(p_f - p_m)^2}{4p(1-p)}$$

where $n_f = 2N_f$ and $n_m = 2N_m$ represent the number of female- and male-derived *sequences* for the locus (N_f and N_m represent the number of individuals sequenced and the factor of 2 arises because of diploidy), and $n_H = 2(1/n_m + 1/n_f)^{-1}$. With approximately equal sample sizes between the sexes ($n_f \approx n_m$, as in the UK Biobank), this reduces further to:

$$\mathrm{E}[\hat{F}_{ST}] = \frac{1}{2n_H} + \left(1 - \frac{1}{2n_H}\right)\frac{(p_f - p_m)^2}{4p(1-p)}$$

which we use in subsequent results. From this last result, it is clear that sampling effects will always contribute somewhat to between-sex divergence in allele frequencies, with the magnitude of sampling effects inversely proportional to n_H.

Beyond sampling effects, the expected allele frequency divergence between the sexes estimated in a sample will increase whenever sex differences in selection generate genuine allele frequency divergence in the population (*i.e.*, $p_f \neq p_m$). Under modest-to-weak selection at a locus (*i.e.*, selection coefficients on the order of 0.1 or less), sex-specific allele frequencies in the population are given by:

$$p_f \approx p + \frac{p(1-p)}{2}\frac{d\ln(\bar{w}_f)}{dp}$$

$$p_m \approx p + \frac{p(1-p)}{2}\frac{d\ln(\bar{w}_m)}{dp}$$

where $\bar{w}_f$ and $\bar{w}_m$ represent the mean relative fitness of each sex with respect to the locus [4]. Note that selection is sexually concordant (SC) when the gradients $d\ln(\bar{w}_f)/dp$ and

$d\ln(\bar{w}_m)/dp$ have same sign; selection is sexually antagonistic (SA) when the gradients have opposite signs. The expected value of the *estimate* of F_{ST} becomes:

$$\mathrm{E}[\hat{F}_{ST}] \approx \frac{1}{2n_H} + \left(1 - \frac{1}{2n_H}\right)\frac{(p_f - p_m)^2}{4p(1-p)}$$

$$\approx \frac{1}{2n_H} + \left(1 - \frac{1}{2n_H}\right)\frac{p(1-p)}{16}\left(\frac{d\ln(\bar{w}_f)}{dp} - \frac{d\ln(\bar{w}_m)}{dp}\right)^2$$

It is clear from the final expression for $\mathrm{E}[\hat{F}_{ST}]$ that any sex difference in selection will, on average, inflate the estimated allele frequency divergence between the sexes (*i.e.*, $\mathrm{E}[\hat{F}_{ST}]$ is inflated whenever $d\ln(\bar{w}_f)/dp \neq d\ln(\bar{w}_m)/dp$).

To illustrate how SA and SC selection affect the correlation between $\mathrm{E}[\hat{F}_{ST}]$ and the minor allele frequency (MAF) per locus, we consider simple models of selection without dominance, with SA polymorphism maintained near equilibrium under balancing selection and SC polymorphism maintained at mutation-selection balance. We subsequently relax the equilibrium assumption via simulation.

The covariance between F_{ST} and MAF under SA selection

Let p refer to the female-beneficial allele at a SA locus ($q = 1 - p$ refers to the male-beneficial allele). With purely additive fitness effects of the alleles, we have:

$$\frac{d\ln(\bar{w}_f)}{dp} = \frac{s_f}{\bar{w}_f}$$

$$\frac{d\ln(\bar{w}_f)}{dp} = \frac{-s_m}{\bar{w}_m}$$

where s_f and s_m represent the selection coefficients for females and males (*i.e.*, the costs to each sex of being homozygous for a SA allele that benefits the other sex). At equilibrium, we have:

$$\frac{d\ln(\bar{w}_f\bar{w}_m)}{dp} = \frac{d\ln(\bar{w}_f)}{dp} + \frac{d\ln(\bar{w}_m)}{dp} = 0$$

$$\frac{d\ln(\bar{w}_f)}{dp} = -\frac{d\ln(\bar{w}_m)}{dp}$$

$$\mathrm{E}[\hat{F}_{ST}] \approx \frac{1}{2n_H} + \left(1 - \frac{1}{2n_H}\right)\frac{p(1-p)}{4}\left(\frac{d\ln(\bar{w}_f)}{dp}\right)^2$$

$$= \frac{1}{2n_H} + \left(1 - \frac{1}{2n_H}\right)\frac{p(1-p)}{4}\left(\frac{d\ln(\bar{w}_m)}{dp}\right)^2$$

Letting $\bar{s} = (s_f + s_m)/2$ and $d_s = s_f - s_m$, at equilibrium we have:

$$p = \frac{1}{2} + \frac{s_f - s_m}{2s_f s_m} = \frac{1}{2} + \frac{d_s}{2(\bar{s}^2 - d_s^2/4)} \approx \frac{1}{2} + \frac{d_s}{2\bar{s}^2}$$

$$d_s = s_f - s_m = \frac{2 - 2\sqrt{1 + (2p-1)^2\bar{s}^2}}{(1-2p)} \approx \bar{s}^2(2p-1)$$

The expected value of the F_{ST} estimate becomes:

$$\begin{aligned}
\mathrm{E}[\hat{F}_{ST}] &\approx \frac{1}{2n_H} + \left(1 - \frac{1}{2n_H}\right)\frac{p(1-p)}{4}\left(\frac{s_f}{1-(1-p)s_f}\right)^2 \\
&= \frac{1}{2n_H} + \left(1 - \frac{1}{2n_H}\right)\frac{p(1-p)}{4}\left(\frac{\bar{s} + \frac{1}{2}d_s}{1-(1-p)\left(\bar{s} + \frac{1}{2}d_s\right)}\right)^2 \\
&= \frac{1}{2n_H} + \left(1 - \frac{1}{2n_H}\right)\frac{\bar{s}^2(1+\bar{s})}{4}p(1-p) + O[(2p-1)^3, \bar{s}^4]
\end{aligned}$$

The final approximation (*i.e.*, neglecting terms of $O[(2p-1)^3, \bar{s}^4]$), which is extremely accurate for $\bar{s} \leq 0.1$, can be used to illustrate how SA selection generates a positive covariance between the minor allele frequency per locus (with MAF = $\min\{p, 1-p\}$) and $\mathrm{E}[\hat{F}_{ST}]$.

If the strength of SA selection ($\bar{s}$) is independent of MAF, then it is clear that $\mathrm{E}[\hat{F}_{ST}]$ for SA loci must increase with MAF. The strength of the positive covariance further increases if $\bar{s}$ and MAF positively covary, as they are predicted to do under models of balancing selection for SA loci. This latter effect arises because conditions for balancing selection at SA loci expand as $\bar{s}$ increases, and the ability of such loci to remain polymorphic (in spite of genetic drift) tend to increase with both $\bar{s}$ and the degree to which the deterministic equilibrium is intermediate. To explore these factors, we modelled the evolution of SA loci evolving under balancing selection and genetic drift. Our subsequent predictions focus on F_{ST} in the population and we neglect effects of sampling. As already noted, error in estimation of allele frequencies inflates estimates of F_{ST}, with the degree of inflation independent of MAF. Since we are interested in the sign of the covariance between $\mathrm{E}[\hat{F}_{ST}]$ and MAF under different forms of selection, our focus on population F_{ST} and its covariance with MAF is sufficient for our purposes, and including sampling effects in the subsequent calculations does not qualitatively change the predictions.

For each locus, we: (*i*) randomly sampled female and male selection coefficients from a uniform distribution between 0 and $s_{\max}$ ($0 < s_{\max} < 1$), where $s_{\max} = 0.01$; (*ii*) retained loci whose sex-specific selection coefficients met conditions for balancing selection (*i.e.*, $\frac{s_f}{1+s_f} <$

$s_m < \frac{s_f}{1-s_f}$; see [5]), and (*iii*) modelled the population allele frequency for the retained SA locus using its stationary distribution [6]:

$$f(p) = \frac{C}{V}\exp\left(2\int \frac{M}{V}dp\right)$$

where M is the expected change in allele frequency per generation at the locus, V is the variance in allele frequency change, and the constant C ensures that the distribution integrates to one.

Assuming there is no dominance in either sex, selection coefficients are small, equal mutation rates per allele, and autosomal linkage, M and V become:

$$M \approx \frac{p(1-p)\left(s_f\bar{w}_m - s_m\bar{w}_f\right)}{4\bar{w}_f\bar{w}_m} + u(1-2p) \approx \frac{s_f s_m}{2}p(1-p)(p^* - p) + u(1-2p)$$

$$V \approx \frac{p(1-p)}{2N_e}$$

where N_e is the effective size of the population, u is the mutation of the locus, p^* is its deterministic equilibrium, and the mean relative fitness of females and males (respectively) are $\bar{w}_f = 1-(1-p)s_f$ and $\bar{w}_m = 1 - ps_m$. The final approximation for M neglects terms of third order in the selection coefficients. Substituting the expressions for M and V, the stationary distribution simplifies to:

$$f(p) = Cx^{4N_e u-1}(1-x)^{4N_e u-1}e^{-N_e s_f s_m (p^*-p)^2}$$

For each locus, we used a rejection sampling algorithm (described in Smith and Connallon [7]) to randomly sample an allele frequency from the stationary distribution for the locus.

F_{ST} for each locus was calculated as:

$$F_{ST} = \frac{\left(p_f - p_m\right)^2}{4p(1-p)}$$

where p_f and p_m correspond to the expected values for sex-specific allele frequencies after selection within the generation:

$$p_f = p + \frac{s_f p(1-p)}{2\bar{w}_f}$$

$$p_m = p - \frac{s_m p(1-p)}{2\bar{w}_m}$$

Representative simulation output for population F_{ST} at SA loci is shown in the following figure, in which we simulated SA loci under three evolutionary scenarios: (1) allele frequency dynamics dominated by genetic drift (left panel, based on the stationary distribution with the exponential term set to one), (2) allele frequency dynamics shaped by selection and drift (middle panel, based on the general stationary distribution); (3) allele frequency dynamics

dominated by selection (right panel, where allele frequencies conform to deterministic predictions). Each panel shows 10^4 simulated loci with minor allele frequency greater than 0.01. For left and middle panels (*i.e.*, non-deterministic scenarios), we set $N_e = 10^6$ and $4N_eu = 0.01$. Each black line shows the least-squares linear regression of F_{ST} on MAF. The results confirm the predicted positive relation between F_{ST} and MAF for polymorphic SA loci.

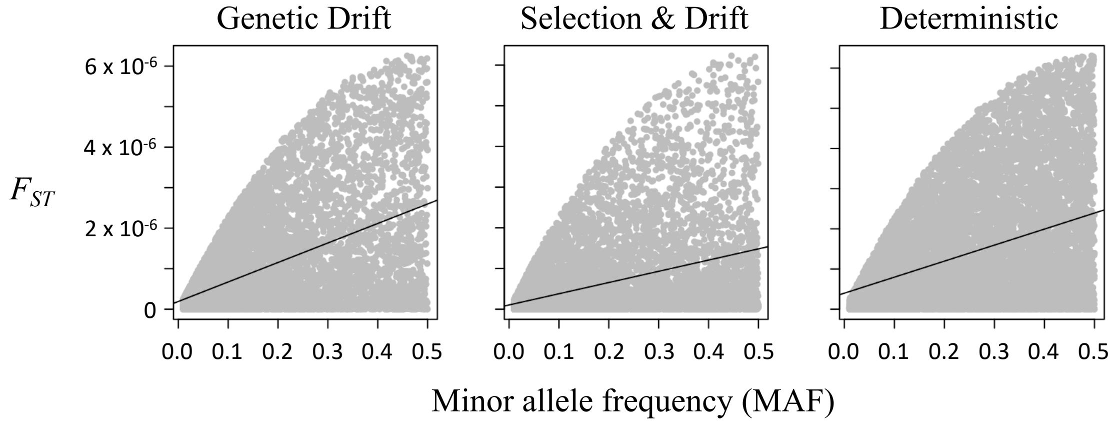

The code and data needed to generate this Figure can be found at https://github.com/filipluca/polygenic_SA_selection_in_the_UK_biobank and https://zenodo.org/record/6824671

The covariance between F_{ST} and MAF under sex differences in purifying selection

For sexually concordant (SC) loci, let p represent the frequency of the deleterious allele at a given locus; t_f and t_m represent the female and male homozygous selection coefficients for the deleterious allele at the locus. Assuming strong selection relative to mutation, and additive fitness effects per locus, the equilibrium allele frequency for a locus with mutation u and parameters t_f and t_m will be $p = 4u/(t_m + t_f)$, leading to the following approximations at mutation-selection balance:

$$\frac{d\ln(\bar{w}_f)}{dp} = -\frac{t_f}{\bar{w}_f} \approx -t_f$$

$$\frac{d\ln(\bar{w}_m)}{dp} = -\frac{t_m}{\bar{w}_m} \approx -t_m$$

$$F_{ST} \approx \frac{p(1-p)}{16}\left(t_f - t_m\right)^2 \approx \frac{p}{16}\left(t_f - t_m\right)^2$$

$$\mathrm{cov}(F_{ST}, MAF) \approx \mathrm{cov}\left(\frac{p}{16}\left(t_f - t_m\right)^2, p\right) \approx \frac{u^2}{4}\mathrm{cov}\left(\frac{\left(t_f - t_m\right)^2}{\bar{t}}, \frac{1}{\bar{t}}\right)$$

where $\bar{t} = \left(t_f + t_m\right)/2$.

Assuming, among loci, that the average difference in selection between sexes scales positively with the strength of purifying selection at each locus—*i.e.*: $\varepsilon = t_f - t_m = x\bar{t}$, where x and $\bar{t}$ are independently distributed random variables, and $\mathrm{E}[x\bar{t}] = \mathrm{E}[x]\,\mathrm{E}[\bar{t}]$ is the expected value of their product—then we have:

$$\mathrm{cov}(F_{ST}, MAF) \approx \frac{u^2}{4}\mathrm{cov}\left(\bar{t}x^2, \frac{1}{\bar{t}}\right) = \frac{u^2}{4}\left(\mathrm{E}\left[\bar{t}x^2\frac{1}{\bar{t}}\right] - \mathrm{E}[\bar{t}x^2]\,\mathrm{E}\left[\frac{1}{\bar{t}}\right]\right)$$

$$= \frac{u^2}{4}(\mathrm{var}[x] + \mathrm{E}[x]^2)\,\mathrm{cov}(\bar{t}, 1/\bar{t})$$

From the final result, we see that F_{ST} will negatively covary with MAF as long as $\mathrm{E}[x^2], \mathrm{var}[\bar{t}] > 0$.

Loci under sex-limited selection represent a special case of the above model. Under male-limited selection, we have $\bar{t} = t_m/2$, $\varepsilon = x\bar{t} = -t_m$, $x = -2$, and therefore:

$$\mathrm{cov}(F_{ST}, MAF) \approx \frac{u^2}{4}(\mathrm{var}[x] + \mathrm{E}[x]^2)\,\mathrm{cov}(\bar{t}, 1/\bar{t}) = u^2\,\mathrm{cov}(t_m, 1/t_m)$$

Likewise, female-limited selection gives us $\bar{t} = t_f/2$, $\varepsilon = x\bar{t} = t_f$, $x = 2$, and $\mathrm{cov}(F_{ST}, MAF) \approx u^2\,\mathrm{cov}(t_f, 1/t_f)$. The final expressions for male- and female-limited loci show that F_{ST} will negatively covary with MAF provided $\mathrm{var}[t_f], \mathrm{var}[t_m] > 0$.

To explore effects of selection and drift on SC polymorphisms, we carried out simulations with allele frequencies drawn from the following stationary distribution:

$$f(p) = \frac{C}{V}\exp\left(2\int\frac{M}{V}dp\right) = cp^{4N_eu-1}(1-p)^{4N_eu-1}e^{-N_e(t_f+t_m)p}$$

where $M = -\frac{1}{4}(t_f + t_m)p(1-p) + u(1-2p)$ and V is the same as above. Initially focusing on the simplest case of sex-limited loci, we sampled selection coefficients per locus from a gamma distribution with shape and scale parameters k and θ, respectively (*i.e.*, $\mathrm{E}[t] = k\theta$ and $\mathrm{var}(t) = k\theta^2$ for the selected sex). Allele frequencies were simulated by rejection sampling (as above; see Smith and Connallon [7]), using the stationary distribution for each locus. For each set of parameters (*i.e.*, N_e, u, k, θ), we generated 5,000 polymorphic loci with minor allele frequencies greater than 1%.

Representative simulation output for population F_{ST} at sex-limited loci is shown in the following figure, in which the two rows show the same data with the *y*-axis in $\log_{10}$ scale (top) and normal scale (bottom). The distribution of fitness effects for the selected sex is assumed to be strongly skewed (gamma shape parameter: k = 1/4), with three average strengths of purifying selection: (1) $N_e\mathrm{E}[t] = 10^4$ (left panel), (2) $N_e\mathrm{E}[t] = 10^3$ (middle panel), and (3) $N_e\mathrm{E}[t] = 10^2$ (right panel). Each panel shows $5\text{x}10^3$ simulated loci with minor allele frequency greater

than 0.01. Parameters include $N_e = 10^6$, and $4N_eu = 0.01$. Each black line shows the least-squares linear regression of $\log_{10}(F_{ST})$ on MAF. The results confirm the predicted negative relation between F_{ST} and MAF for loci under sex-differential purifying selection.

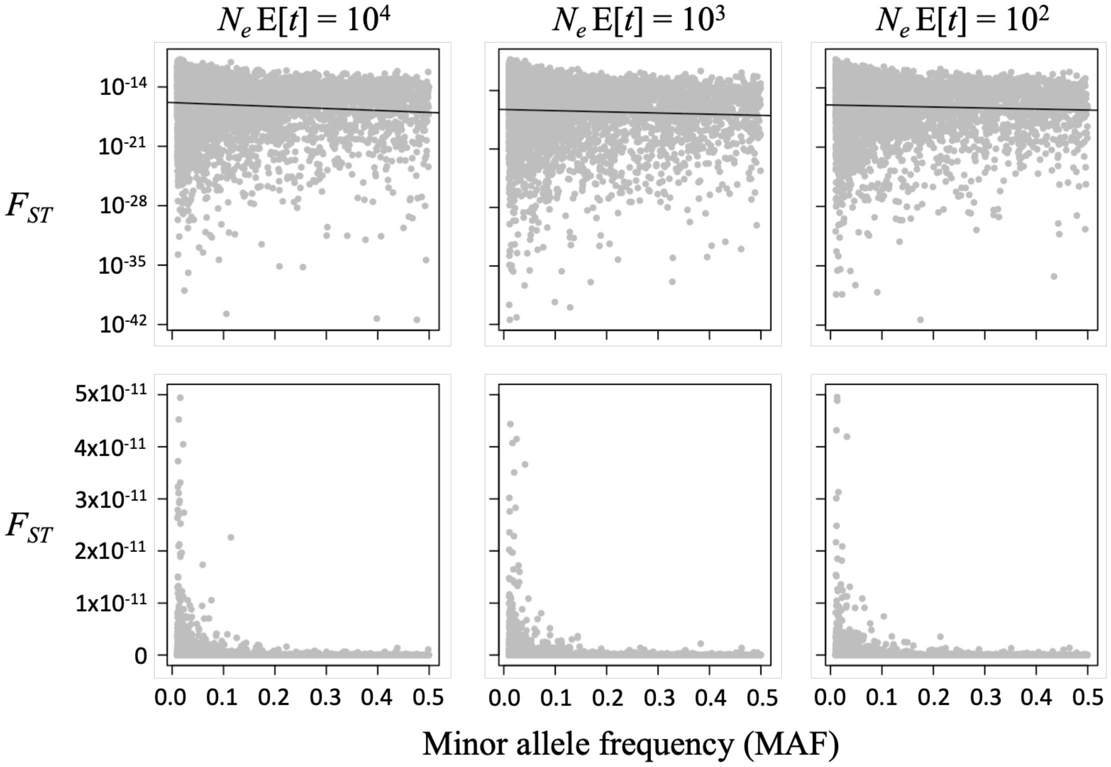

The code and data needed to generate this Figure can be found at https://github.com/filipluca/polygenic_SA_selection_in_the_UK_biobank and https://zenodo.org/record/6824671

We then explored the more realistic scenario in which there is a mixture of sex-limited loci and loci that affect the fitness of both sexes, with f_{SL} representing the proportion of loci that are sex-limited. We defined whether a given locus was sex-limited by sampling a random variable from a Bernoulli distribution with success probability of f_{SL}. Selection coefficients for loci with sex-limited effects were randomly drawn from a gamma distribution, as described above. For loci affecting the fitness of both sexes, we generated selection coefficients in each sex by randomly sampling from a symmetric bivariate gamma distribution, with a cross-sex genetic correlation of r_{mf} (the algorithm for pseudo-random sampling of correlated selection coefficients from a bivariate gamma distribution is presented in Morrow and Connallon [8]). Allele frequencies for each locus were simulated using a rejection sampler based on the

stationary distribution for the locus (*i.e.*, given its selection coefficients). For each set of parameters (*i.e.*, N_e, u, f_{SL}, k, θ, r_{mf}), we generated 5,000 polymorphic loci with minor allele frequencies greater than 1%. The following figures show results with $r_{mf} = 0.9$ and $N_e = 10^6$, and plausible distributions of fitness effects. Between-sex F_{ST} negatively covaries with MAF for every parameter combination that we examined. Overall, models of SC genetic polymorphism consistently predict a negative covariance between MAF and between-sex F_{ST}.

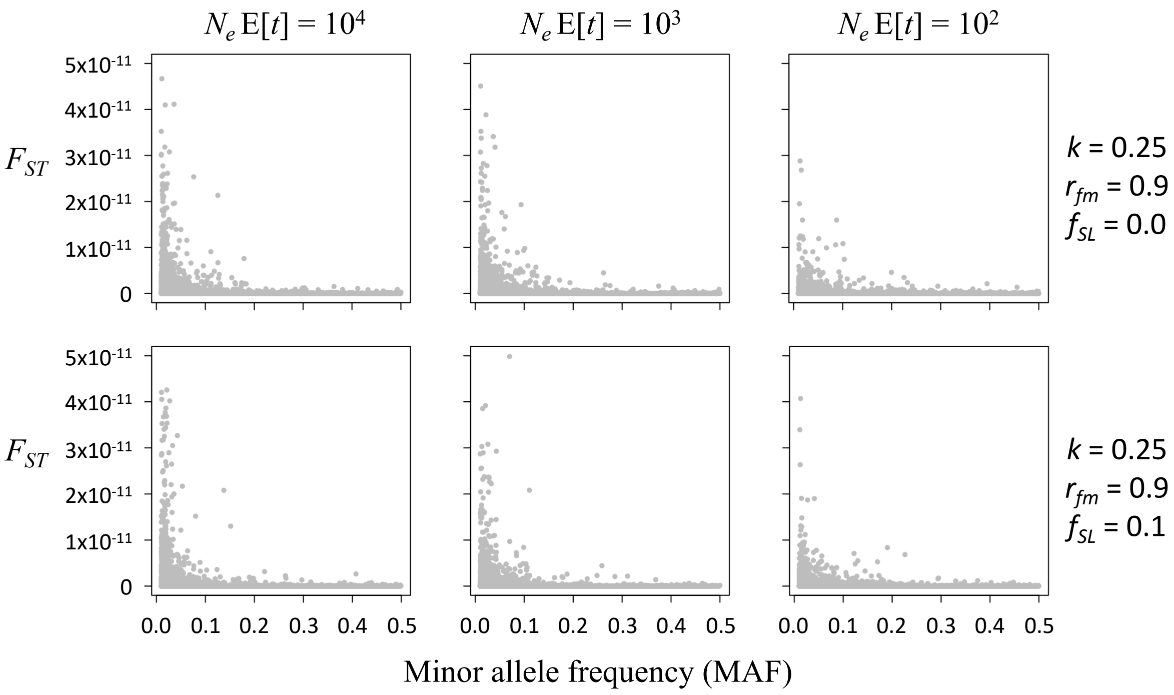

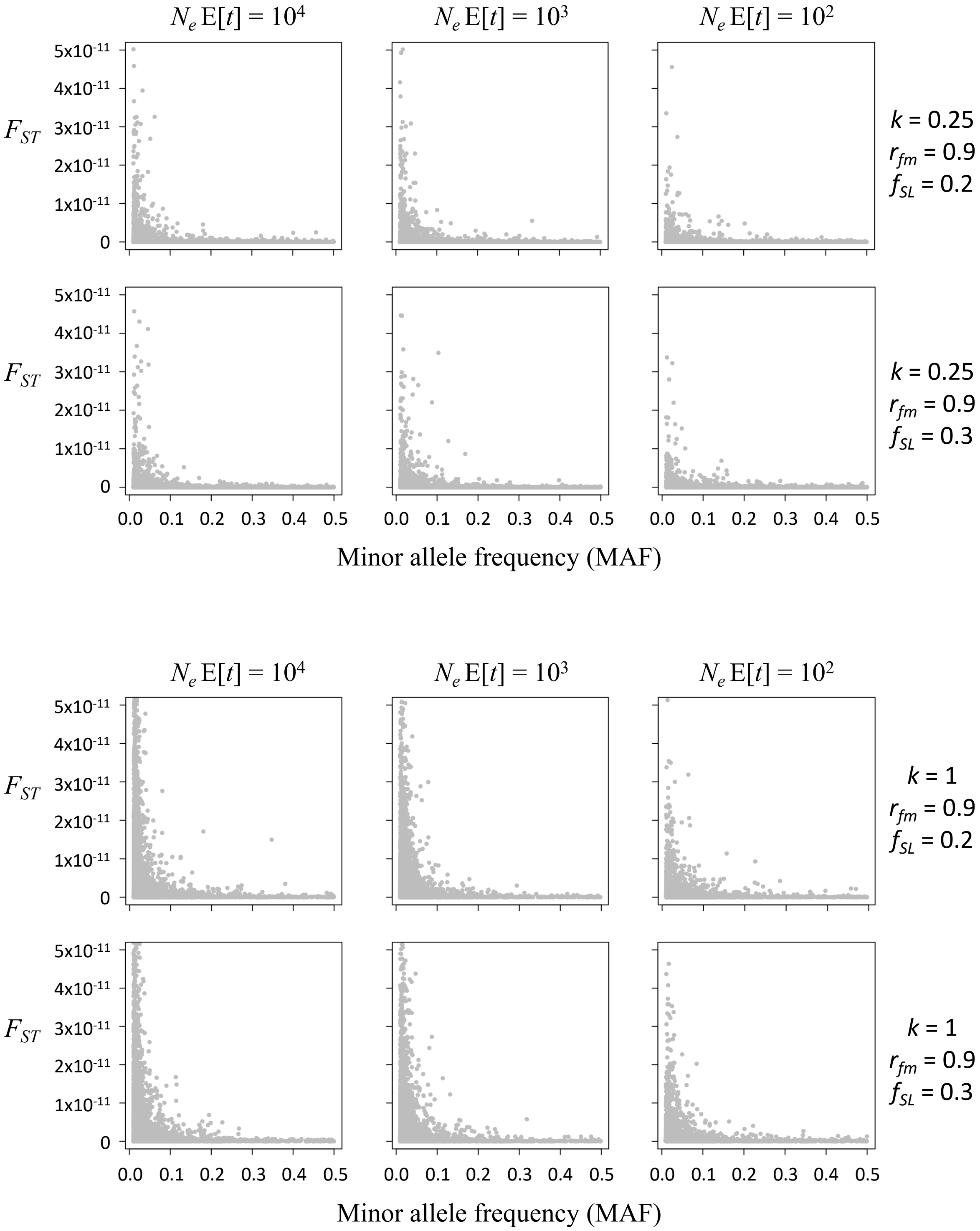

The code and data needed to generate this Figure can be found at https://github.com/filipluca/polygenic_SA_selection_in_the_UK_biobank and https://zenodo.org/record/6824671

SI Appendix, Section H: Associations between metrics of sex differences in selection and MAF

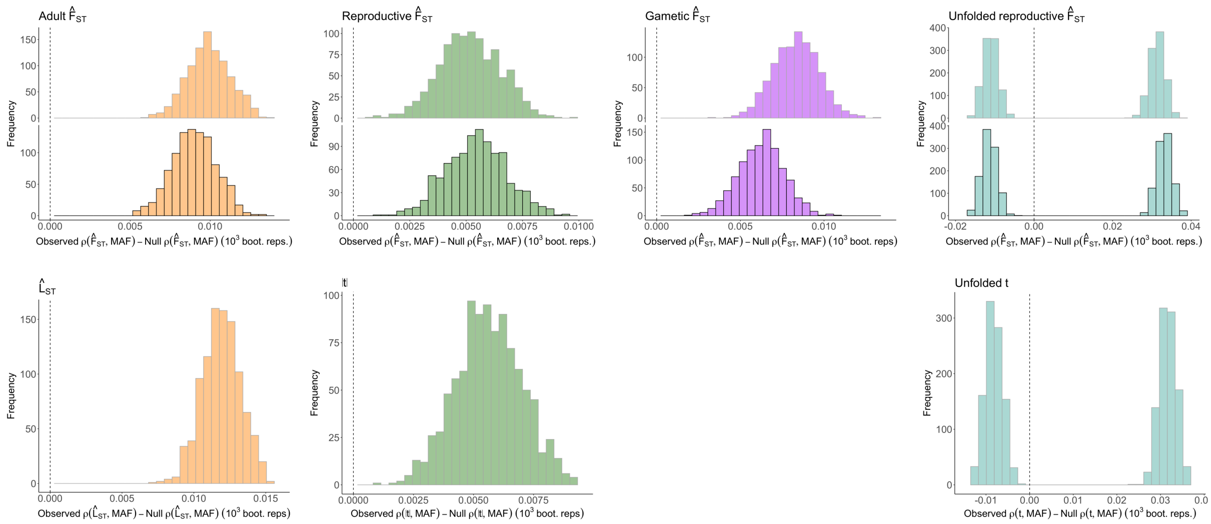

Fig. SH1. Difference between the Spearman's rank correlation in the observed and null data, across 1,000 bootstrap replicates, where the correlation is between metrics of sex differences in selection and MAF. In each panel, grey-outline histograms (top) represent the difference between observed and empirical null data, while black-outline histograms (bottom) represent the difference between observed and theoretical null data (there is no theoretical null for mixed-model metrics, so only grey-outline histograms can be presented); vertical line intersects 0 (no difference between observed and null). All bootstrap replicates are greater/smaller than zero for relevant comparisons and metrics, so empirical p-values are all <0.001. The code and data needed to generate this Figure can be found at https://github.com/filipluca/polygenic_SA_selection_in_the_UK_biobank and https://zenodo.org/record/6824671

SI Appendix, Section I: Associations between metrics of sex differences in selection and candidates for balancing selection

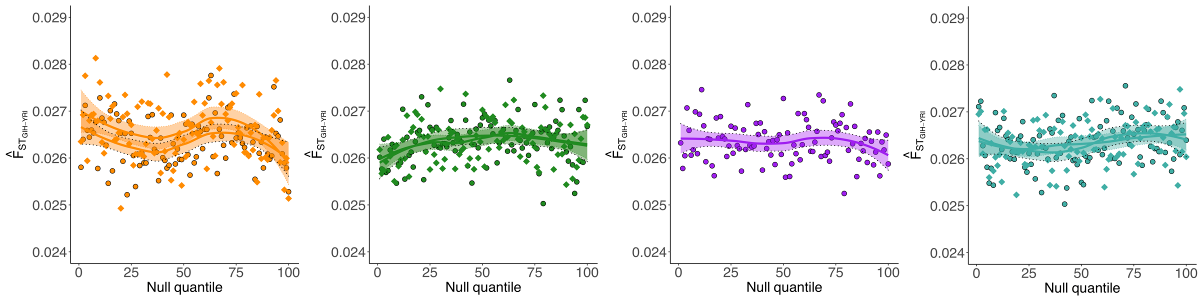

Fig. SI1. Associations between metrics of sex differences in selection and between-population (GIH-YRI) F_{ST} estimates. Between-population $\hat{F}_{ST}$ is presented across 100 quantiles of the null for each metric of sex-differential selection. Each panel corrects for ascertainment bias of allele frequencies among highly sex-differentiated sites (*i.e.*, Fig. 6A-D). For visualisation purposes, this was done by averaging, in each quantile, between-population $\hat{F}_{ST}$ across 20 quantiles of MAF in the UK Biobank (such that UK Biobank MAF is approximately equal across quantiles). The code and data needed to generate this Figure can be found at https://github.com/filipluca/polygenic_SA_selection_in_the_UK_biobank and https://zenodo.org/record/6824671

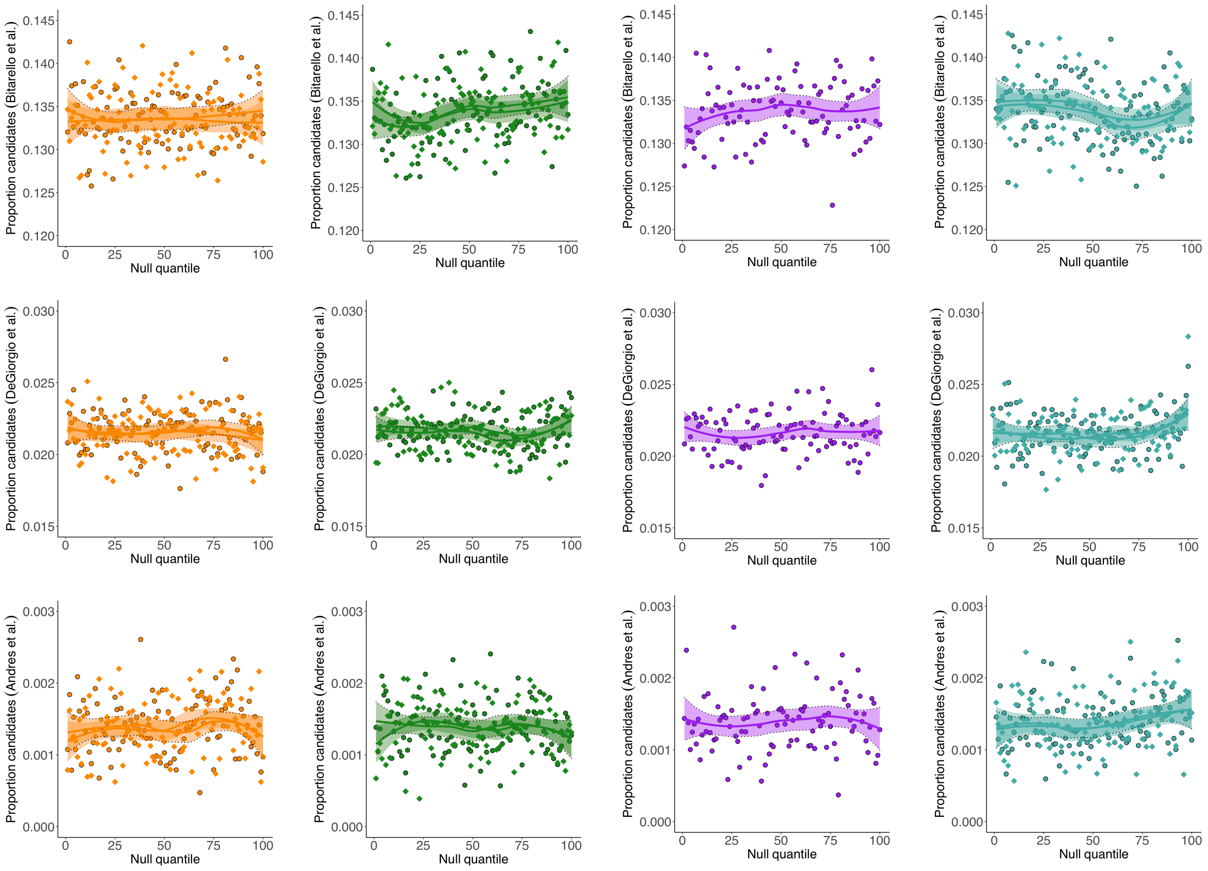

Fig. SI2. Associations between metrics of sex differences in selection and candidates for balancing selection. The proportion of sites that overlap with candidates for balancing selection from previous studies (Bitarello et al. 2018 [9], top row; DeGiorgio et al. 2014 [10], middle row; Andrés et al. 2009 [11]; bottom row) is presented across 100 quantiles of the null for each metric of sex-differential selection. Each panel corrects for ascertainment bias of allele frequencies among highly sex-differentiated sites (*i.e.*, Fig. 6A-D). For visualisation purposes, this was done by averaging, in each quantile, the proportion of sites that overlap with candidates for balancing selection across 20 quantiles of MAF in the UK Biobank (such that UK Biobank MAF is approximately equal across quantiles). The code and data needed to generate this Figure can be found at https://github.com/filipluca/polygenic_SA_selection_in_the_UK_biobank and https://zenodo.org/record/6824671

Tab. SI1. Effect sizes and p-values for associations between metrics of balancing selection and metrics of sex-differential selection. Effect sizes are linear regression coefficients (for between-population F_{ST} estimates and Tajima's D), Spearman's rank correlations (for allele age) and log-odds ratios (for candidates for balancing selection). No p-values are significant after FDR multiple-testing correction across metrics.

METRIC	Adult F_{ST}	L_{ST}	Reproductive F_{ST}	|t|	Gametic F_{ST}
**Between-population F_{ST}**	$\beta=-0.000$ (p=0.879)	$\beta=-5.793\times10^{-3}$ (p=0.026)	$\beta=0.000$ (p=0.743)	$\beta=0.002$ (p=0.631)	$\beta=0.002$ (p=0.227)
**Tajima's D (YRI)**	$\beta=-0.004$ (p=0.015)	$\beta=-4.436\times10^{-3}$ (p=0.139)	$\beta=0.001$ (p=0.677)	$\beta=0.004$ (p=0.248)	$\beta=-0.001$ (p=0.652)
**Tajima's D (GIH)**	$\beta=-0.001$ (p=0.737)	$\beta=1.131\times10^{-3}$ (p=0.791)	$\beta=0.005$ (p=0.028)	$\beta=0.008$ (p=0.160)	$\beta=0.004$ (p=0.088)
**Allele age**	$\rho=-0.000$ (p=0.401)	$\rho=-0.002$ (p=0.021)	$\rho=0.000$ (p=0.697)	$\rho=-0.000$ (p=0.940)	$\rho=0.001$ (p=0.190)
**Candidates (Bitarello et al. 2018)**	$\beta=0.000$ (p=0.662)	$\beta=-5.995\times10^{2}$ (p=0.873)	$\beta=-0.004$ (p=0.024)	$\beta=0.008$ (p=0.080)	$\beta=0.000$ (p=0.708)
**Candidates (Andrés et al. 2009)**	$\beta=-0.016$ (p=0.391)	$\beta=-3.508\times10^{4}$ (p=0.326)	$\beta=-0.019$ (p=0.306)	$\beta=-0.023$ (p=0.599)	$\beta=-0.008$ (p=0.651)
**Candidates (DeGiorgio et al. 2014)**	$\beta=-0.006$ (p=0.207)	$\beta=-3.573\times10^{3}$ (p=0.686)	$\beta=0.005$ (p=0.254)	$\beta=-0.003$ (p=0.773)	$\beta=0.003$ (p=0.547)

Tab. SI2. Effect sizes and p-values for associations between metrics of balancing selection and metrics for distinguishing the form of sex-differential selection. Effect size definitions are the same as in Table SI1. Note that positive values of unfolded F_{ST} do not directly relate to the extent of sex-differentiation (i.e., large values can either signal sex-differential SC selection, or SC selection of equal magnitude in both sexes). Bolded values indicate significance (q<0.05) after FDR multiple-testing correction across metrics.

METRIC	Unfolded F_{ST} (negative values)	Unfolded t (negative values)	Unfolded F_{ST} (positive values)	Unfolded t (positive values)
**Between-population F_{ST}**	β=–0.007 (p=0.041)	β=–0.009 (p=0.012)	β=0.006 (p=0.030)	β=0.007 (p=0.036)
**Tajima's D (YRI)**	β=0.001 (p=0.779)	β=–0.000 (p=0.990)	β=–0.007 (p=0.002)	β=–0.006 (p=0.014)
**Tajima's D (GIH)**	β=–0.001 (p=0.908)	β=–0.000 (p=0.983)	β=–0.009 (p=0.006)	β=–0.007 (p=0.033)
**Allele age**	**ρ=–0.004 (p=0.007)**	ρ=–0.003 (p=0.043)	**ρ=–0.005 (p<0.001)**	ρ=–0.004 (p=0.004)
**Candidates (Bitarello et al. 2018)**	β=0.001 (p=0.766)	β=0.001 (p=0.916)	β=0.011 (p=0.014)	β=0.012 (p=0.010)
**Candidates (Andrés et al. 2009)**	β=–0.003 (p=0.950)	β=0.015 (p=0.766)	β=0.015 (p=0.706)	β=0.040 (p=0.252)
**Candidates (DeGiorgio et al. 2014)**	β=–0.006 (p=0.623)	β=–0.010 (p=0.396)	**β=0.079 (p<0.001)**	**β=0.079 (p<0.001)**

References

1. Ruzicka F, Dutoit L, Czuppon P, Jordan CY, Li X, Olito C, et al. The search for sexually antagonistic genes: Practical insights from studies of local adaptation and statistical genomics. Evol Lett. 2020;4: 398–415. doi:10.1002/evl3.192
2. Connallon T, Jordan CY. Accumulation of deleterious mutations near sexually antagonistic genes. G3. 2016;6: 2273–84. doi:10.1534/g3.116.031161
3. Finucane HK, Bulik-Sullivan B, Gusev A, Trynka G, Reshef Y, Loh PR, et al. Partitioning heritability by functional annotation using genome-wide association summary statistics. Nat Genet. 2015;47: 1228–1235. doi:10.1038/ng.3404
4. Connallon T, Sharma S, Olito C. Evolutionary consequences of sex-specific selection in variable environments: Four simple models reveal diverse evolutionary outcomes. Am Nat. 2019;193: 93–105. doi:10.1086/700720
5. Kidwell JF, Clegg MT, Stewart FM, Prout T. Regions of stable equilibria for models of differential selection in the two sexes under random mating. Genetics. 1977;85: 171–183.
6. Crow JF, Kimura M. An Introduction to Population Genetics Theory. Harper and Row; 1970. doi:10.2307/1529706
7. Smith SRT, Connallon T. The contribution of the mitochondrial genome to sex-specific fitness variance. Evolution. 2017;71: 1417–1424. doi:10.1111/evo.13238
8. Morrow EH, Connallon T. Implications of sex-specific selection for the genetic basis of disease. Evol Appl. 2013;6: 1208–1217. doi:10.1111/eva.12097
9. Bitarello BD, de Filippo C, Teixeira JC, Schmidt JM, Kleinert P, Meyer D, et al. Signatures of long-term balancing selection in human genomes. Genome Biol Evol. 2018;10: 939–955. doi:10.1093/gbe/evy054
10. DeGiorgio M, Lohmueller KE, Nielsen R. A model-based approach for identifying signatures of ancient balancing selection in genetic data. PLoS Genet. 2014;10:

e1004561. doi:10.1371/journal.pgen.1004561

11. Andrés AM, Hubisz MJ, Indap A, Torgerson DG, Degenhardt JD, Boyko AR, et al. Targets of balancing selection in the human genome. Mol Biol Evol. 2009;26: 2755–2764. doi:10.1093/molbev/msp190
