# Supplementary material for: Polygenic signals of sex differences in selection in humans from the UK Biobank
Source: PLoS Biol. 2022 Sep 6;20(9):e3001768. doi: 10.1371/journal.pbio.3001768 (PMC9481184; doi:10.1371/journal.pbio.3001768)
Supplement: Attachment — Submitted filename: Ruzicka et al. revised Reply to reviewers.docx [file pbio.3001768.s003.docx]

To the editors,

First, we would like to apologise for the delay in submitting our revised manuscript. We were keen to address the comments as thoroughly as possible, but the UK Biobank is a massive dataset and re-analyses are inevitably time-consuming. This has also been a particularly busy (not to mention COVID-delayed) period for the three authors. Second, we wish to thank the four reviewers and editor for their thoughtful and helpful comments. As you will see, we have extensively revised the manuscript in light of these comments. We have re-written large portions of the manuscript to clarify our intent, re-analysed our data upon reviewer suggestions, and developed new theory and empirical analyses to strengthen previous arguments.

Because the response document is rather lengthy, we briefly summarise the main features of our revision:

1. It was noted that our analyses could at times be more robust. To address this, we have made the following changes:
   a. For all empirical analyses, we now focus exclusively on LD-pruned data (R3 suggestion). Assumptions of independence in statistical tests are thus more likely to be met, increasing the accuracy of effect sizes and p-values that we report. Main conclusions remain unchanged.
   b. We now emphasise mixed-model results for detecting sex-differential selection (R2 and R3 suggestion). Fig. 4 presents exclusively mixed-model tests, and analyses of biological functions and modes of evolution (Figs. 5 and 6) incorporate mixed-model metrics. We have nonetheless retained the Fst-based focus in the main text, for reasons that we expand upon in response to specific comments below.
   c. We now use LD-score regression to examine associations between metrics of sex-differential selection and functional annotations (R2 suggestion) and traits (R3 suggestion). We also use it to establish that signals are polygenic (following a comment by R4). Our conclusions remain qualitatively unaltered.
   d. We now investigate the association between metrics of sex-differential selection and Tajima’s D—a metric of genetic diversity that carries some signal of different scenarios of selection (R1 suggestion). These new analyses complement previous analyses of allele age, between-population Fst and candidates for balancing selection.
2. It was noted that our interpretations could at times be more cautious. Throughout the manuscript—the Discussion especially—we have substantially modified our language and we now present a more detailed and cautious set of interpretations of our findings. For example, we clarify: (i) predictions for the association between Fst and MAF in the absence vs. presence of sex-differential selection (R1 and R4 comments); (ii) why sex-differentiation with respect to non-reproductive components represents equivocal evidence for sex-differential selection (R2 and R3 comments); (iii) what we can and cannot conclude based on the positive associations between Fst and MAF (R2 and R3 comments); (iv) when our analyses support “sex-differential” broadly vs. “SA” selection specifically (R3 comment).
3. R1, R2, and R4 highlighted an unexplained discrepancy between permuted and theoretical nulls for reproductive Fst. We have now resolved this discrepancy. Put simply, our previous theoretical null did not adequately account for variation in the sample sizes of genotyped sites, along with site-specific variability in the estimates of sex-specific mean and variance for fitness, and Hardy-Weinberg deviations. The heterogeneity between loci arising from missing data led to some inflation of the permuted null data (which were sensitive to these features of the data) relative to the theoretical null predictions. Accounting for these sources of variation removes any difference between theory and permuted nulls.
4. We have strengthened the argument that SA selection—as opposed to just “sex-differential” selection—is occurring. Previously, we supported our argument for SA selection based on the observed positive association between MAF and between-sex Fst metrics. We have now developed a new metric which allows us to partition signals of sex-differential selection into SA and sexually concordant components. This new metric—“unfolded reproductive Fst” and its mixed-model equivalent “unfolded t”—quantifies the product of variant effects on LRS in each sex, showing that both sexually concordant *and* SA selection contribute to signals of sex-differentiation affecting reproductive success.

Note that in the revised document with track changes, heavily-modified areas of the manuscript have been highlighted in red.

Overall, our manuscript presents the most robust evidence yet for sex-differential, including SA, genomic variation in humans. Our revised manuscript also clearly highlights the pros and cons of each new test, facilitating appropriate application of our analysis strategy to new datasets. For these reasons, we hope our manuscript will now be acceptable for publication in *PLOS Biology*.

Sincerely,

The authors

————————————————————————————————————————

Dear Dr Ruzicka,

Thank you for submitting your manuscript "Polygenic signals of sexually antagonistic selection in contemporary human genomes" for consideration as a Research Article at PLOS Biology. Your manuscript has been evaluated by the PLOS Biology editors, an Academic Editor with relevant expertise, and by four independent reviewers.

IMPORTANT: We'd like to apologise for an unfortunate glitch in our process. When we called in the additional metadata in preparation for peer review, our template letter invited you to include any previous reviews from other journals. You did so, correctly, but our system should have then alerted us to this fact, at which point we would have taken those reviews into account, potentially contacting the other journal, and then highlighting this fact to our reviewers. Unfortunately this alert failed, and your paper went out to new reviewers, but including your reviews and rebuttal. You will see that most of the reviewers nevertheless acknowledge this fact, and have commented accordingly. Overall, we hope that this process will prove useful, despite not providing the expedited journey that we intended. I'll apologise similarly to the reviewers for the confusion.

When I described the situation to the Academic Editor, s/he said "I hope that you can convey that [the required revision] is at the milder end of the spectrum. It has been reviewed already, and several of the new reviewers noted that they were therefore reluctant to require yet more analyses. The suggested analyses make sense to me, and would be helpful, but I think that the main issue is to present the work more cautiously, which should be straightforward." I hope that this additional advice will be helpful when deciding how to revise your manuscript.

In light of the reviews (below), we will not be able to accept the current version of the manuscript, but we would welcome re-submission of a much-revised version that takes into account the reviewers' comments. We cannot make any decision about publication until we have seen the revised manuscript and your response to the reviewers' comments. Your revised manuscript is also likely to be sent for further evaluation by the reviewers.

————————————————————————————————————————
Reviewer #1:

[identifies himself as Daniel Berner]

Comments on PBIOLOGY-D-21-02588R1, 'Polygenic signals of sexually antagonistic selection in contemporary human genomes'

I have digested this study as a new reviewer, and to save time, I have haphazardly but not systematically checked how the authors addressed previous reviewer criticism.

This study uses a very large human data set, including whole-genome genotype data and information on offspring number, to look for genome-wide signatures of sexually antagonistic (SA) selection. The key analyses include the evaluation of the observed distribution of intersex differentiation (Fst) against null expectations, the association of Fst with MAF, the location of candidate SA SNPs in (non)functional genomic regions, and looking for evidence of balancing selection at candidate SA SNPs. The study performs quite rigorous data filtering to reduce the risk of detecting spurious SA signals, and the theoretical framework underlying their analyses is very carefully developed in my view. The key findings include a collective (but of course not SNP-level) pattern consistent with SA selection on both survival and reproductive output emerging from several analyses, and that SA candidate loci are enriched for functional sites. The study finds no evidence, however, that SA candidate loci are under balancing selection.

The quest for demonstrating SA selection is currently an active field in biology, so the study is certainly topical. Moreover, I feel that methodologically, the study is quite rigorous and in many aspects goes beyond what has previously been presented in the field. Of course there have been (more or less problematic) previous attempts to detect SA selection in humans, so this work is not addressing an entirely novel question or producing earth-shattering results. Nevertheless, the overall quality of the investigation and evidence seems outstanding to me, and I feel it is a strength, not a weakness, to revisit important existing research problems with new data and methods. Also, the writing is very clear and analyses are well justified and explained; I really enjoyed reading this manuscript and feel the investigation is well suited for publication in PLoS Biology.

Thank you for the very helpful review.

I consider this work in very good shape (it is a revision, after all) and have only relatively minor comments. Perhaps the most serious would be to consider testing for elevated genetic diversity around the SA candidates, as a complementation to the balancing selection analysis. Here the details, not sorted by relevance:

1) Line 89 (and L139-140 etc): I was first puzzled by this association between Fst and MAF being taken as evidence of SA selection, because for the allele frequency difference, a positive correlation to MAF exists under pure neutrality (because low-MAF polymorphisms cannot yield high differentiation; Roesti et al. 2012 BMC Evol Biol). I thus performed a brief simulation experiment on the association between differentiation and MAF in the absence of SA selection. This indicated that indeed the raw intersex allele frequency difference is correlated positively to MAF, but Fst is not. Given that the Fst-MAF correlation is just one of the signals taken as evidence of SA selection by the authors, and that its theoretical basis is well developed in the Methods section, I trust this conclusion, although it goes somewhat beyond my direct intuition.

(See a related comment by R4). In a heavily revised Results and Discussion, we now clarify the distinction between predictions for the Fst vs. MAF correlation in the absence and presence of sex-differential selection.

In the absence of contemporary sex-differential selection (i.e., when the fitness effects of variants are equal between the sexes, or when variants are neutral), we expect no correlation between Fst and MAF (as noted also in Ruzicka et al. 2020 Evol. Lett.). Indeed, this is one argument for using Fst rather than raw allele frequency difference as a metric of differentiation.

In the presence of contemporary sex-differential selection, we expect positive correlations between Fst and MAF when: (i) variation evolved under neutrality in the past; (ii) variation evolved under balancing selection in the past; (iii) variation has recently reached intermediate frequencies due to a partial or incomplete selective sweep. We expect negative associations between Fst and MAF when variation evolved under purifying selection in the past. Overall (as we now clarify in the manuscript), the MAF vs. Fst association allows us to narrow down the set of scenarios that can plausibly contribute to the patterns we observed.

2) L114: This passage makes me wonder about loci that may have antagonistic fitness effects within a sex across the life cycle. E.g., loci that may increase female survival but reduce female fertility. Is this possibility addressed in the paper (I may have overlooked)?

(See also a similar comment by R2). We agree that looking for antagonistic fitness effects across the life cycle is interesting. In response to this comment:

1. We now quantify the relationship between different fitness components by calculating genetic correlations, using LDscore regression. These analyses (Fig. 5A) show that there is no significant correlation between sex-differential viability effects (e.g., adult Fst or Lst) and sex-differential reproductive effects (e.g., reproductive Fst or |t|). The absence of a significant correlation may indicate no antagonistic pleiotropy or—more likely—a similar fraction of variants with synergistic and antagonistic effects on different fitness components (see point 2).
2. We have developed theoretical models for detecting antagonistic pleiotropy under the assumption that contributing loci have sex-limited effects. Our preliminary analyses based on these models suggest that antagonistic pleiotropy does indeed occur in the UK Biobank (see SI Appendix A, not for publication).

However, these analyses are not, in our opinion, suitable for publication as part of this manuscript. This is because predictions about the genetic basis and evolutionary histories of SA variants are distinct from predictions about within-sex AP variants. Moreover, the manuscript already includes a large number of analyses relating to SA selection. A stand-alone manuscript that builds on our preliminary analyses of AP is a more suitable medium for properly exploring this question.

3) L189 and elsewhere: I think in line with what professional statisticians recommend, the term 'statistically significant' should be discarded (e.g., Wasserstein et al. 2019, American Statistician). This dichotomous label is not needed; can simply give the P-value, or even better, the parameter estimates along with their confidence intervals.

Done.

4) L296-302: I think a signature consistent with balancing selection would be elevated genetic diversity in the close (i.e., tightly physically linked) vicinity of the candidate SA polymorphisms. I encourage the authors to explore this. For instance, take your best few hundred or so SA candidate SNPs, and retrieve all polymorphisms occurring within windows of, say, 4 kb around them. Then do the same for a large number of non-SA-candidate SNPs. A prediction would be that around the SA candidate SNPs, genetic variation is elevated. Not sure what would be the best diversity metric, but Haenel et al. (2019, Evolution Letters) suggest that the density of high-MAF SNPs is a particularly sensitive diversity measure.

In addition to our previous analyses of allele age, between-population Fst, and previous candidates for balancing selection, we have now performed an analysis of windowed genetic diversity (we used Tajima’s D as the statistic in question). Results of this analysis are consistent with previous analyses, with little association between metrics of sex-differential selection and Tajima’s D (once ascertainment bias due to elevated MAF in the UK Biobank is controlled for; see SI Appendix, Section I).

5) L351-355: You here argue that sample size could be an issue, but how large are the Biobank Japan and Finngen data bases?

Biobank Japan and Finngen databases contain N=178,242 and N=150,831 individuals, respectively. These databases are large but still smaller than those where signals of sex-differential viability selection have been reported, including here (N~249,000) and by Pirastu et al. 2021, Nat. Gen. (N~500,000 and N~2,500,000, respectively). Therefore, a signal in the larger datasets could have arisen from ascertainment biases introduced by active participation (as Pirastu et al. argue), or more power to detect a signal of sex-differential viability selection (as we highlight). Moreover, differences in the extent of sex-differential viability selection between populations could also contribute to differences in detected signals. In short, we need UK Biobank-sized datasets with passive participation (i.e., random sampling of individuals) to definitively establish the presence of sex-differential viability selection.

In a heavily-revised Discussion, we have now added further information on the size of the Biobank Japan and Finngen databases. We have also clarified that signals of sex-differential selection captured in adult Fst and Lst metrics do not represent the strongest evidence for SA selection in the UK Biobank. Rather, analyses of reproductive fitness components (along with additional analyses in the manuscript) are more compelling on this particular point.

6) L424: These (or analogous) predictions for mapping artifacts have also been described in previous studies, which could perhaps be acknowledged:

<https://doi.org/10.1186/1745-6150-7-17>
https://[doi.org/10.1111/1755-0998.12613](http://doi.org/10.1111/1755-0998.12613)
https://[doi.org/10.3390/genes](http://doi.org/10.3390/genes)10040320
https://[doi.org/10.1111/mec.15255](http://doi.org/10.1111/mec.15255)

Thanks for the references. We have added the first three to our reference list (the fourth was present in the original version of our manuscript).

7) L439, ‚eliminate mapping errors'. Perhaps tone this down slightly; using such a threshold-based approach, it is unlikely that errors are completely eliminated, as some errors may just pass below the detection criterion.

Amended to “reduce the chance of”.

8) L473: Put 'proxy' in singular.

Done.

9) Figure 2A-C: How about adding a LOESS smoother to the observed data?

Done.

Also, in B, is there an explanation as to why the permuted data also seem to show an excess of SNPs in the top Fst quantiles?

(See similar comment by R2 and R4) We have resolved the discrepancy between permuted and observed null. Previously, the theoretical null assumed no variation in per-locus sample sizes, which leads to locus-specific variability in fitness data and estimated deviations from Hardy-Weinberg equilibrium genotype ratios. Not accounting for this feature of the dataset caused an inflation of the empirical null relative to the theoretical null.

Accounting for locus-specific sample sizes, fitness data, and estimated deviations from Hardy-Weinberg ratios fully resolves the difference between permuted and theoretical null distributions for reproductive Fst (i.e., the two distributions are statistically indistinguishable).

10) L902 and 915: I generally think that a lot of significance testing we report is unnecessary. Here, for instance, the histograms convey the full information, no need for P values in my view.

We agree that effect sizes and confidence intervals are more informative than P-values. Nonetheless, P-values may provide information in cases where it is unwieldy to present histograms and confidence intervals (e.g., genetic correlation plots). We therefore retain effect sizes and confidence intervals (where constraints of visual presentation allow it) and P-values as well.

————————————————————————————————————————
Reviewer #2:

The work has been reviewed before by three reviewers and so I will comment on the responses to these first.

Thank you for highlighting further areas of improvement in our manuscript.

1. Generally, the authors have responded well to the comments of Reviewer #1 and Reviewer #2.

2. I agree with the previous reviewers regarding the fact that the mixed-model association FST values should be the focus of the manuscript. It would streamline the presentation, especially of the results, which remain hard to follow, and as the authors state in their response to Reviewer #3, it is these estimates that have the best chance to control for population artefacts (though see below).

We agree that mixed-model metrics are more robust for establishing structure-corrected signals of sex-differential selection (see similar comment by R3). For further emphasis, we now present the results of mixed-model tests as a stand-alone figure in the Results (now Fig. 4), rather than just the Supplementary Materials. We have also simplified the presentation of the Results by focussing on establishing signals of sex-differential selection first, and then establishing the form of sex-differential selection, rather than combining both into one long theory section and another long empirical section.

Nonetheless, we have retained Fst metrics in the main text for the following reasons:

1. Part of our paper’s aim is to present a framework for future studies of sex-differential selection. Using Fst allows us to partition the life-cycle into its components, whereas mixed-model metrics do not (e.g., there is no mixed-model equivalent of “gametic Fst”.)
2. Fst metrics are amenable to the development of formal theoretical null models. By contrast, there is no theoretical null model for the relevant mixed-model metrics, and the sources of differentiation in the absence of sex-differential selection are therefore less clear.
3. Focussing on Fst metrics places our paper in a rich historical tradition of population differentiation tests that will be familiar to most evolutionary biologists, which should increase the dissemination of our work. Moreover, we are able to highlight the pros and cons of Fst and mixed-model metrics, such that researchers will be more aware of each metric’s respective limitations.

3. I do not feel they have addressed the concern of Reviewer #3 in their final major point regarding the breakdown of the data into signals of viability and reproductive fitness.

(See also a similar comment by R1). Regarding investigating signals of antagonistic fitness effects across the life cycle, we now quantify the relationship between different fitness components by calculating genetic correlations between components, using LDscore regression. These correlations (Fig. 5A) show that there is no significant correlation between sex-differential viability metrics (e.g., adult Fst or Lst) and sex-differential reproductive metrics (e.g., reproductive Fst or |t|). The absence of a significant correlation may indicate no antagonistic pleiotropy or—more likely—a similar fraction of variants with synergistic and antagonistic effects on different fitness components.

To further investigate the extent of sex-limited antagonistic pleiotropy, we have developed models and performed preliminary analyses which suggest that antagonistic pleiotropy does indeed occur in the UK Biobank (see SI Appendix A, not for publication). However, these analyses are not, in our opinion, suitable for publication as part of this manuscript because predictions about the genetic basis and evolutionary histories of SA variants are quite distinct from predictions about AP variants. Moreover, the manuscript already includes a large number of analyses relating to SA variants, and would become unwieldy if analyses of AP variants were to be included. A stand-alone manuscript that builds on our preliminary AP analyses of antagonistic pleiotropy would a more suitable medium for such an examination.

4. The authors need to conclusively demonstrate why there is a differenc between the theoretical and empirical nulls, rather than providing conjecture that it could stem from deviations from HWE.

(See also similar comments by R1 and R4). The reason for the discrepancy was due to not accounting for a variety of factors which collectively generated a discrepancy between theoretical and empirical nulls. In particular, the theoretical null ignored variation in per-locus sample sizes, which leads to locus-specific variability in fitness data and estimated deviations from Hardy-Weinberg equilibrium genotype ratios. Not accounting for this feature of the dataset caused an inflation of the empirical null relative to the theoretical null.

We now only focus on adults with valid LRS data in our calculations of pm, pf, pm’ and pf’. Accounting for locus-specific sample sizes, fitness data, and estimated deviations from Hardy-Weinberg ratios fully resolves the difference between permuted and theoretical null distributions for reproductive Fst (i.e., the two distributions are statistically indistinguishable).

I am loathe to propose further work given that the manuscript has already been under review. However, I strongly feel that there are some key issues that remain unresolved. The authors claim that this is "the first study to present unambiguous signals of sex-differential selection in human genomes" and they claim to show wide-spread, polygenic, sex-differential selection that has a major impact on genomic variation. This is a fairly bold claim and as such requires stronger evidence than I feel has been presented here.

1. I am concerned that there is the potential for sample ascertainment to give extensive bias. The UK Biobank is a healthier sample than the general UK population and the sample is only 46% male. So what causes females to be more likely to register to the UK Biobank? Are there any traits with a genetic basis that could be associated with this ascertainemnt, which then translates ascertainment into sex biases at the frequencies of different alleles. I suspect so, and there is nothing one can do about it. This issue goes beyond discussing this as a potential caveat. As the authors make extrodinary claims - that sexual antagonism maintains variation at a substantial number of locations across the genome (i.e. it is polygenic) - there is a requirement for more evidence then a single association study. I would be more convinced if they replicated the top associations within another biobank.  I also feel they need to show that ascertainment of individuals could not cause the results observed here. They also need to control for age-at-enrolment within their analyses.

2. Does between-sex FST increase with the age group of the participants? Women have a higher life expectancy and so will be over-represented in the older age classes.  LRS, as measured by number of children, has varied greatly due to cultural factors over the past decades, with family sizes generally becomming smaller over time. As women simply survive longer and are more likely to enter the study in later-life, could these patterns not simlply represent cultural changes across generations linked to viability selection?

The reviewer raises a number of good points.

First, we completely agree that allele frequency differentiation between adults is not definitive evidence for sex differences in selection. As the reviewer correctly points out (see a similar comment by R3), the active sampling procedure used in the UK Biobank dataset means that there are multiple reasons why males and females could show allele frequency differences (e.g., sex-differential cultural factors, sex differences in propensity to undertake such studies, sex differences in the age of participants). Although we can correct for some confounders explicitly (e.g., the age of participants and assessment centre in our Lst analyses), we cannot correct for them all. As such, we cannot conclusively distinguish between competing interpretations for between-sex allele frequency differentiation among adults, including sex-differential participation bias (Pirastu et al’s interpretation) or sex-differential viability selection. To resolve the issue, we need a UK Biobank-size dataset, sampled through passive rather than active participation. No randomly-sampled dataset of equal or greater size to the UK Biobank is yet available.

We now highlight (in both the Abstract and Discussion) the issue of ascertainment bias and the more compelling signals of sex-differential selection in analyses of reproductive success variation (which avoid the issue of ascertainment bias; see below). These changes should better emphasise points that were perhaps understated in the previous version of the paper, and readers should have less room for misconceptions.

Second, we emphasise that much stronger evidence for sex-differential selection in the UK Biobank is derived from our LRS analyses. This is because LRS-based metrics control for allele frequency differences among adults and, in effect, control for associated biases (e.g., sex differences in propensity to participate in the study, age, cultural factors, artefacts due to read mis-mapping). Indeed, the fact that the UK Biobank contains LRS data informed our choice to study this biobank in the first place. It is another reason why we cannot adequately replicate our analysis strategy in another biobank (e.g., FinnGen and Biobank Japan do not contain LRS data).

Third, sexual antagonism being “polygenic” is not, in our opinion, an extraordinary claim. There is ample evidence to support this idea: (i) the theoretical conditions for polygenic SA variation—i.e., sex discordant effects of mutations and/or sex discordant selection—are unrestrictive and well documented (see Connallon and Clark 2014, Proc B); (ii) there is previous evidence for sex-discordant selection on individual traits (most notably, height) in the UK Biobank and other contemporary human population samples (see Stearns et al. 2012, Proc B, and Sanjak et al. 2017, PNAS) and these traits are known to be polygenic; (iii) new analyses explicitly partition signals of sex-differential selection affecting reproductive success into SA and sexually concordant components. These new analyses show that both types of variant—not just sex-differential SC variants—contribute to overall signals.

We agree, however, that polygenic SA selection need not “maintain variation” in the sense that it generates long-term balancing selection. Conditions favouring long-term balancing selection, especially in a small-Ne species like humans, are indeed restrictive. This point has been emphasised in the revised manuscript.

3. The authors should use LDScore regression to calculate the correlation between LRS and FST from the mixed model summary statistics. Also as a way to test for enrichment using annotation groups. This would be far more robust (controlling for intercept terms which may reflect population stratification) than the t-statistics presented here.

We agree that LDscore regression is a more robust way to test for enrichment using annotation groups than the genic enrichment method we presented previously. We now use it to test for such enrichments.

We cannot use LDscore regression to correlate LRS and Fst because the former is an individual-level metric and the latter is a population-level metric (i.e., every individual has an estimate of LRS but it does not have an Fst estimate).

4. The null model for LRS should not be based on randomised trait values within each sex but rather based on a simulated heritable trait with equal mean and variance across the sexes and genetic correlation = 1.

It is not clear why our permutation procedure for reproductive Fst is inappropriate. Our procedure ensures that neither sex differences in the allele frequencies among adults, nor sex differences in the relationship between genotype and LRS, contribute to variation in reproductive Fst in the permuted data, leaving only estimation error to contribute Fst variation to the permuted data.

We also use LDscore regression to quantify overall signal in our metrics (including LRS-based metrics). Each metric shows significantly greater-than-zero SNP-heritability in observed data but approximately zero heritability in permuted data (SI Appendix, Section D).

5. Please also fit PCs of the chromosomes as fixed effects when calculating the LOCO regression coefficients. BoltLMM controls for general population stratification on chromosomes other than the focal, but not the one in which the marker effect size is being estimated.

Done.

————————————————————————————————————————
Reviewer #3:

Ruzicka et al. develop a suit of methods to test for contemporary sexually-antagonistic (SA) viability selection—using allele frequency differences between males and females in a large cohort—and reproductive selection—using the number of children reported for said individuals. They apply these methods to the UK Biobank and reject a null model of zero SA selection in this cohort.

The paper has many notable strengths: Readers will benefit from the inclusion of a theoretical expectation and the permutation-based null alongside it; the control for population structure is novel (though the authors, unfortunately, do not ask whether it successfully removed some of the stratification due to participation bias that Pirastu et al., Nature Genetics 2021 reported); the estimates of SA selection that they derive will be useful for the community and the analysis is one of the most thorough I have seen in this space. The breakdown into the fitness components is cool.

Thank you for the very thorough review.

At the same time, key choices made by the authors need to be revisited in order for the manuscript to merit publication. Most importantly, these include presenting the most conservative version of their analyses in the main text rather than in the supplement—in particular setting a proper null expectation and control for LD in all analyses. If these issues, as detailed below, can be solved, I think the work would make for a valuable contribution to the literature.

The authors do well to clarify that they are not estimating the strength of sexually antagonistic selection (SAS) or its importance in shaping patterns of genetic variation. Rather, they set more modest goals of testing a null hypothesis of no contemporary SA selection, and pointing to the likeliest candidate targets of SAS. Given this fact, however, I would encourage the authors to show an LD-pruned and population structure-aware version of their tests as main figures and text. The current presentation of the results in the main text is anti-conservative, as it is inflated by both factors.

These are all good points. We now focus all analyses on LD-pruned data. Though LD-pruning also increases the risk of false negatives (which we were also keen to avoid), our statistical tests may indeed have been anti-conservative because they assumed independence between loci.

We also agree that mixed-model metrics are more robust for establishing structure-corrected signals of sex-differential selection (see a similar comment by R2). For further emphasis, we now present the results of mixed-model tests as a stand-alone figure in the Results (Fig. 4), rather than the Supplementary Materials (as we did last time); they are also presented as part of Figs. 5 and 6.

Nonetheless, we have retained Fst metrics in the main text for the following reasons:

1. Part of our paper’s aim is to present a framework for future studies of sex-differential selection. Using Fst allows us to partition the life-cycle into its components, whereas mixed-model metrics do not (e.g., there is no mixed-model equivalent of “gametic Fst”.)
2. Fst metrics are amenable to the development of formal theoretical null models. By contrast, there is no theoretical null model for the relevant mixed-model metrics, and the sources of differentiation in the absence of sex-differential selection are therefore less clear. At minimum, the correspondence between empirical and theoretical null data for Fst provides an appealing reality check that we understand the scope of estimation error in these data.
3. Focussing on Fst metrics places our paper in a rich historical tradition of population differentiation tests that will be familiar to many evolutionary biologists. This is likely to increase the dissemination of our work. Moreover, we are able to highlight the pros and cons of Fst and mixed-model metrics, and researchers will therefore be more aware of each metric’s respective limitations.

At several points along the paper, the authors seem to implicitly assume "all or nothing" theoretical models or interpretation that weaken their analyses and conclusions somewhat. One example is in the relationship reported for minor allele frequency and Fst (around line 212)—a part I might, as the previous cycle's reviewer 1 had suggested, either remove or further flesh out possible interpretations and caveats to the conclusion. An example caveat is ascertainment bias. Do the authors observe similar relationships with allele frequency in a different sample (with relatively diverged ancestry)?

The reviewer has highlighted two important limitations:

1. The possibility that ascertainment bias may drive signals of between-sex allele frequency differences among adults in the UK Biobank in the absence of genuine sex-differential viability selection (See a similar comment by R2). This is something we cannot correct for entirely (though we correct for some aspects of this, e.g. including age as a covariate in analyses of Lst). Conclusive evidence awaits replication in a Biobank-scale sample obtained through passive rather than active participation. At present, no other sample is available which contains both genotype and LRS data, on a similar scale to the UK Biobank. Both of these conditions are needed to replicate the analyses we have carried out and produce interpretable conclusions (e.g., failing to replicate the signal in a smaller dataset would be inconclusive).
2. The assumption that the extent and form of sex-differential selection is temporally stable. One argument to support this is that proxies for fitness in this population correlate positively with proxies for fitness in the recent past (Zietch et al. 2014, PNAS). However, given drastic cultural changes in humans over the last thousands of years, whether proxies for fitness in contemporary populations correlate positively with fitness over much longer timescales (i.e. timescales that are relevant to affecting the MAF spectrum) is all but untestable. This same limitation applies to studies that use the relationship between GWAS effect size and MAF to test for purifying selection on individual traits (e.g., Zeng et al. 2018, Nat. Gen.)

While these issues were mentioned in our original Discussion, we now further mention them in the Abstract and in a heavily revised Discussion.

Another example is in the overarching assumption that alleles are either experience the same selective pressures in males and females or are subject to SA selection. What about the (seemingly more likely, agnostically) case of sex-specific selection, in which selective effects differ to some unknown extent between the sexes?

We agree that our uses of the terms “sex-differential selection” and “SA selection” were at times ambiguous. We now clarify when our tests are indicative of sex-differential selection (including SC and SA selection), and when they are indicative of SA selection to the exclusion of SC selection. We also divide the Results into one section where the aim is to test a “no sex-differential selection” null. Then, in a second section, we investigate the form of sex-differential selection (Fig. 3), supporting the presence of SA polymorphisms. Finally, given that a large fraction of the manuscript is focussed on sex-differential selection rather than SA selection specifically, we have changed the title of the paper to reflect this.

Yet another example of the "all or nothing" interpretive approach is in a section entitled "Evolutionary Analysis of candidate SA SNPs" (lines 282-302. The authors test for a correlation between measures of balancing selection and between-sex Fst. We might, at best, learn that there is no evidence for a very strong hypothesis of a monotonic relationship between contemporary SA selection and long-term balancing selection—but this is a rather weak statement, and one that the authors still need to put in substantial work to establish. (Because, as in other parts of the paper, there is no proper control for LD or consideration of estimation noise).

All analyses now correct for LD through LD-pruning.

As regards the section titled “evolutionary analyses of candidate SA SNPs”, we have now reframed this section to include more tests of the mode of evolution affecting SA loci. Specifically, we now include analyses of the relationship between MAF and Fst—which suggest that the evolutionary dynamics of SA loci are shaped by drift, sweeps and/or balancing selection—in this section. Placing the previous analyses of long-term balancing selection in this broader context should help clarify, as the reviewer correctly points out, that our hypothesis tests for long-term balancing selection were quite specific.

The authors also innovate in their control for population structure stratification. They replace Fst with the test statistic Lst, the log-odds ratio of an allele being carried by a male relative to a female, beyond what can be explained by the main axes of genetic ancestry (i.e., by including principal components of the genotype matrix as covariates). Is this approach enough to remove some of the stratification reported by Pirastu et al.? Or does everything that follows boil down to differences in interpretation of the same signals, where Ruzicka et al. interpret them as SA selection and Pirastu et al. interpret them as artefacts due to participation bias? Readers would benefit from some discussion on this.

(See a similar comment by R2) The active participation sample in the UK Biobank dataset means that there are multiple reasons why males and females could show allele frequency differences among adults (e.g., cultural factors, differences in propensity to undertake such studies, age, etc.). Although we can attempt to correct for some potential confounders explicitly (e.g., we correct for the age of participants and assessment centre in our Lst analyses), we cannot conclusively distinguish between sex-differential participation bias and sex-differential viability selection in driving between-sex allele frequency differentiation relating to non-reproductive fitness components. Pirastu et al. 2021 (Nat. Genet.)—who interpret allele frequency differentiation among adults as the result of sex-differential participation bias—similarly cannot exclude the possibility of sex-differential viability selection in driving such differentiation.

This limitation of both ours and Pirastu et al’s study is covered at greater length in the revised Discussion.

The analysis of correlation with GWAS effect estimates seems invalid to me, because of the same two familiar reasons: It does not set up a null, empirical or otherwise, and does control for LD in any way. For the latter, if complex traits like testosterone are the trait of interest, genetic correlations can be tested for rather than raw correlations.

We now control for LD. Instead of our original approach, we now use LDscore regression to estimate genetic correlations between metrics of sex-differential selection and traits.

Speaking of T, the observation "Loci associated with T had lower than average gametic Fst" needs to be fleshed out: There are no statistics to back it up, and the authors do not suggest what it might mean. This remains especially unclear to me given that variation in testosterone levels have highly distinct genetic bases in the two sexes (Flynn et al., Eur J Hum Genetics 2021; Sinnott-Armstrong, Naqvi et al., eLife 2021).

Upon re-analysis using genetic correlations estimated through LDscore regression, there are no longer any significant associations with testosterone.

Minor comments:

-       Can the authors cite sources supporting the choice of cutoff age of 45 for child bearing / having? Also, does the UKB questionnaire ask only about biological children?

The cut-off of 45 years was chosen for two reasons. First, the yearly number of children for individuals (especially women) after the age of 45 is small (roughly ~20 births per year for females based on ONS data, given ~0.3% of births are to women greater than 45 years of age, ~650,000 yearly births in the UK, and that ~1% of all UK individuals aged between 45-69 are included in the UK Biobank dataset). Second, Sanjak et al. (2017, PNAS)—which is an important and relevant precedent to our study—used this cut-off as well. We have now included some more details on this point in the revised Materials and Methods.

The UKB questionnaire asks about “Number of children fathered” (men) and “Number of live births” (women). Although it is conceivable that this would have been mis-interpreted to include non-biological children, this would again affect a very small fraction of the dataset. We clarify that this is a possible source of mis-reporting.

-       In the UKB (an order of a million chromosomes sampled at each site), a 1% cutoff means essentially no rare variation will be observed, further weakening the conclusion of the analysis on the relationship between minor allele frequency and SA selection.

We agree that excluding rare sites is more likely to exclude sexually concordant than SA variants. However, this does not hinder the interpretability of the relationship between Fst and MAF. Rather, the relationship is interpretable for variants >1% frequency, but not for variants <1% frequency.

————————————————————————————————————————
Reviewer #4:

This review is incomplete (due to a mistake on the reviewer's part about timing of the deadline), so some aspects of the how the methods yielded the results observed may not have been fully considered by the reviewer.

General comment (written quickly this morning):

I really liked this paper -- I think it shows a conservative approach for testing for the effect of sexually antagonistic selection in humans, which is an area of considerable interest. The authors seem to have gone to extensive lengths to try to control a range of sources of error, considering other previous analyses and implementing controls from them. If anything, I think some of these controls may cause an underestimation of the effects. As with anything highly polygenic and driven by subtle allele frequency changes, I am still not sure there isn't some unconsidered variable driving these observations, but I have been mulling it over for a few weeks now and nothing obvious would make sense. Thus, I think it's a good candidate for PLOS Biology. Well written and clear.

Thank you for the very helpful review.

Other comments organized from previous notes:

Major comments:

1. Any explanation for why the difference between observed and theoretical is so much larger than observed and null for panel 2E? The other ones look like good agreement between theoretical and null distributions. This one is looking at the difference between adult and offspring allele frequencies in male vs. difference in females. I was surprised by the differences in this figure and felt like I would have liked some more explanation about why that might occur.

(See similar comment by R1 and R2). Previously, the theoretical null assumed no variation in per-locus sample sizes, which lead to locus-specific variability in fitness data and estimated deviations from Hardy-Weinberg equilibrium. These factors would tend to artificially inflate the empirical null relative to the theoretical null, potentially accounting for the discrepancy.

We now only focus on adults with valid LRS data in our calculations of pm, pf, pm’ and pf’, as well as accounting for variation in per-locus fitness data and estimated deviations from Hardy-Weinberg equilibrium. Doing so resolves the difference between permuted and theoretical null data for reproductive Fst.

Minor comments:

Line 159: Is it really true that intermediate frequency alleles inflate FST_hat more than low frequency alleles under drift? I thought the whole point of FST is that it's representing drift irrespective of allele frequency. Not sure about this particular point.

(See similar comment by R1) The reviewer is correct that Fst_hat and MAF are independent, under drift and in the absence of sex-differential selection.

However, when there is sex-differential selection in the contemporary population, a positive correlation between Fst_hat and MAF can occur when: the frequency spectrum of the variants reflects a history of neutral evolution, or a history of balancing selection, or alleles contributing to differentiation are in the process of sweeping. Given each of these scenarios for the allele frequency spectrum, genuine sex-differential selection in the contemporary population will generate a positive association with MAF.

We have clarified predictions for the relationship between Fst_hat and MAF in the revised manuscript.

Line 174: I can appreciate that to be conservative and make it harder to detect SA selection, you need to adopt these filtering criteria, but couldn't differences in missing rates be caused by an indel that has been favoured in one sex? Would this increase chance of false negatives? Or are these really egregious bionformatic errors? I guess the method described on line 425-441 is thought to only exclude loci under implausibly strong SA selection? (given human mortality data)?

Our choice to use conservative artefact-filtering measures to establish signals of SA selection was informed by two factors. First, convincing evidence for SA selection in human genomic data is lacking. Second, methods to distinguish bioinformatic errors from signals of sex-differential selection are still in development (i.e., very few studies focus on this issue specifically, with Kasimatis et al. 2021, Genetics and Bissegger et al. 2021, Mol. Ecol. being the two most relevant precedents). We agree that in principle—and almost certainly in practice—this conservative approach may exclude genuine sex-differentially selected loci, such as indels.

We emphasise the risk of increased false negatives and our justification for choosing a conservative approach in the revised Materials and Methods.

Figure 2: I can appreciate that you'd like to maintain the same colour scheme for panels D-F as in A-C, but I didn't read the caption carefully at first and I thought that the open bars were somehow representing the null and coloured bars representing the data. If you could somehow more clearly emphasize on the Figure itself that these are representing the difference between observed - null and observed - theoretical, that would help. I think making both hollow and filled bars the same colour would help me notice this more easily. Maybe add a legned with a hollow box and a filled box?

We have changed the colouring scheme in Fig. 2D-F (and similar plot types) to avoid confusion.

Line 205: I'm not sure that this is evidence about polygenic. If we knew nothing about linkage, it could be possible that all the enriched high-FST SNPs occurred in a single block of highly linked alleles with one causal allele under strong selection that simply wasn't quite enough to get above the bonferroni.

Agreed. We now provide additional support for this argument by using LDscore regression to quantify the association between a genomic region’s “LDscore”—the extent to which genetic variants tags other variants due to LD—and the SNP-heritability explained by genetic variants. Under a polygenic model, SNPs in high-LD regions should have higher SNP-heritability because they are more likely to tag causal variants. This analysis reveals consistently positive associations between LDscore and SNP-heritability (SI Appendix, Section D), which is most simply interpreted as evidence for polygenicity.

Line 262: what are the effect sizes here? With these types of tests, significant p-values can be found with very small effects, as the sample size is very large. Can you provide some estimate of odds ratio or something? (how much more likely is a high FST to be found in a genic vs. non-genic region).

Line 269: It wasn't totally clear to me what was being done here without going to the methods, so a little more explanation would help. What is the assocation that is being tested on line 272? Is this testing somehow whether the high FST SNPs are enriched among the GWAS candidate loci? It's explained clearly on line 555 but would be easier to understand with a bit more detail in the results where it's first introduced.

Line 280: Interesting that loci affecting testosterone had lower than average FST -- but I guess maybe that's a conflict that has already been resolved to the sex chromosomes?

The analyses mentioned in each of the above comments have been replaced with an analogous but more robust analysis approach—LDscore regression—following suggestions by R2 and R3.

As regards functional analyses, we now present effect size estimates derived from LDscore regression (Fig. 5B). The effect size in question is “enrichment”—namely, the fraction of SNP-heritability of explained by a given category of site (e.g., coding regions) for a given metric of sex-differential selection, divided by the fraction of all SNPs that occur in that same category.

As regards genetic correlations between metrics of sex-differential selection and individual traits, we have now clarified how such correlations should be interpreted in the Materials and Methods. Specifically, we have polarised metrics of sex-differential selection, such that positive genetic correlations denote that trait-increasing alleles have, on average, female-beneficial effects (more precisely, trait-increasing alleles have more positive effects on females than males for the metric in question). Conversely, negative genetic correlations denote that trait-increasing alleles have, on average, male-beneficial effects.

As a result of re-analysis using LDscore regression, the significant correlation with testosterone in our original analysis is no longer significant.

Line 368: Interesting that there was no signature of balancing selection on these loci -- reasons outlined here are appropriate.
