# Supplementary material for: Polygenic signals of sex differences in selection in humans from the UK Biobank
Source: PLoS Biol. 2022 Sep 6;20(9):e3001768. doi: 10.1371/journal.pbio.3001768 (PMC9481184; doi:10.1371/journal.pbio.3001768)
Supplement: Attachment — Submitted filename: Ruzicka et al. revised Reply to Reviewers 2.docx [file pbio.3001768.s004.docx]

Reviewer #3:

[identifies himself as Arbel Harpak]

Overall, I think the authors did an excellent job at addressing reviewers' comments seriously and thoroughly. I include a few comments / questions I am curious about below, but strongly believe that at this point the authors have earned the right to leave a small fraction of (now seven!) reviewers' musings unattended…

Thanks again for taking the time to review our manuscript and make thoughtful comments.

1. I did not understand the response to my last comment asking why alleles with maf <1% were excluded. I think that in practice examining only very common alleles may give a warped view of selection.

In our previous response, we meant that we are making inferences about the properties of sex-differentiated sites with MAF>1%, and that our inferences about such sites are reasonable.

We fully agree that sites with MAF<1% are likely to have properties that differ from those with MAF>1%. We have chosen to restrict our analyses to sites with MAF>1% because such sites are less likely to contain artefacts and more likely to carry genuine signal of sex differences in selection, as oppose to largely sampling error.

We have clarified our rationale for choosing the MAF>1% cut-off in the revised Methods.

2. I remain curious about the weird correlation of SD-selection statistics with testosterone (or, in the current version, lack of correlation). A seemingly very related 2022 bioRxiv preprint by Zhu et al. examined the relationship between sex differences in genetic effects on traits and male-female Fst. Only one trait repeatedly (in three datasets; though much smaller than the UKB) came up as slightly significant---testosterone. Can the authors comment on whether or not this is discrepant with their results, and why? On that note, perhaps some of the GWAS analyzed in Fig. 5C should be repeated with a sex-stratified GWAS. In particular extremes like testosterone where genetic effects on the trait are highly sex-specific.

Our revised genetic correlation analysis showed that there *is* a significant correlation between testosterone and the mixed-model metric of sex-differential viability selection (see Fig. 5C). Zhu et al., who focus on signals of sex-differential viability selection, also find an association with testosterone, and replicate our results. There is therefore no discrepancy to be resolved.

We thank the reviewer for highlighting this result. We now make reference to it, and the associated manuscript, in our revised Discussion.

3. I appreciate the efforts by the authors to make the language and the presentation more careful, but I think there's still some room for improvement. One example: The legend of figure 5 says that "positive correlations indicate that "a trait is more beneficial in females than in males". This is both ill-defined (I believe the authors you mean higher trait values are favored) and overstates. The statement can be made about the benefit of alleles associated with the trait; but given plausible complications, such as pleiotropy, recruitment biases… I think any claim from a single-trait test that does not consider correlations between traits, levels of background selection, recruitment or other modes of population stratification is at best suggestive about the focal trait being under selection. The authors should try and be extra cautious when it comes to claim about sex-specific selection, especially when they themselves fully acknowledge the looming caveat of study

recruitment biases.

We agree that a single-trait test cannot be used to definitively establish that a trait is more beneficial in one sex than the other, for the reasons outlined by the reviewer and expanded upon in our Discussion. Because it is merely suggestive evidence, we have now changed “imply that a trait is more beneficial…” to “suggest that a trait is more beneficial…”. Readers are then referred to our Discussion where the caveats are discussed in greater detail.

Moreover, we have amended our text from “a trait is more beneficial to females than in males” to “higher trait values are more beneficial to females than males”. This statement is accurate because a positive correlation between adult Fst and a trait need not imply that selection favours elevated trait values (as implied the reviewer’s suggestion). For example, a positive correlation between adult Fst and height could imply that selection favours smaller males than females, but net selection for height across both sexes could be positive, negative, or zero (depending on the relative strength of selection in each sex).
