# Supplementary material for: Polygenic signals of sex differences in selection in humans from the UK Biobank
Source: PLoS Biol. 2022 Sep 6;20(9):e3001768. doi: 10.1371/journal.pbio.3001768 (PMC9481184; doi:10.1371/journal.pbio.3001768)
Supplement: Attachment — Submitted filename: Ruzicka et al. Response to Previous Reviewer comments.docx [file pbio.3001768.s002.docx]

**This manuscript was previously reviewed at *PNAS*. Below, we include the full set of comments by the three reviewers of our original paper (blue text) and our responses to these comments (black text).**

**Because the document is rather extensive, we preface the more detailed responses by briefly highlighting responses to the three major concerns raised by the reviewers:**

1. **Reviewer #1 was concerned that uncontrolled linkage disequilibrium (LD) between neutral variants and causal SA variants would undermine our main conclusion that there is a polygenic signal of sex differences in selection in the human genome. We now clarify that LD, by itself, cannot explain the excess differentiation between the sexes that we report (i.e., the excess beyond effects of statistical sampling, population structure and technical artefacts, which we explicitly control for), and that sex differences in selection *must be invoked* to explain the pattern. While the excess differentiation is almost certainly attributable to some combination of SNPs responding directly to selection (causal SNPs) and SNPs responding indirectly by association with causal SNPs, this doesn’t undermine the test for a polygenic signal. Nevertheless, we have carried out the additional analysis suggested by the reviewer, using a subset of LD-pruned SNPs, and find (as expected, but also comfortingly) that our original results are replicated in the LD-pruned set. In short, the main argument of our manuscript—that SA polymorphisms leave a detectable population genomic signal in the human genome—is unaffected by LD.**

**We elaborate on some of the finer points and implications of LD for our study in a more detailed response to Rev. 1 (below).**

2. **We originally stated that the positive association between Fst and MAF that we report is an “unambiguous signal” of SA polymorphism. All three reviewers argued that differences between current forms of selection (which affect Fst) and past forms of selection (which affect MAF) may generate a positive association in the absence of SA polymorphism. We agree that some alternative models of selection—e.g., temporally fluctuating selection with sex differences in the strength but not the direction of selection—could, in principle, lead to a positive association between Fst and MAF. We have consequently tempered our language and clarified that there is some remaining uncertainty regarding the specific forms of sex-differential selection that could plausibly explain the patterns that we report.**

**There are several reasons why we favour our original interpretation regarding SA selection: (i) SA selection is a simpler model for the pattern that we report than the scenarios proposed by the reviewers (though we agree that they are plausible); (ii) SA selection is a more efficient mechanism for generating allele frequency differences between sexes compared to scenarios involving selection in the same direction but with different strengths in each sex (i.e., all else equal, the effect of a single SA locus on allele frequency divergence between the sexes will be at least two-fold greater than a locus responding to the same direction of selection in each sex); (iii) there is compelling prior evidence for SA selection at the phenotypic level in the UK Biobank (Sanjak et al. 2017, *PNAS*), consistent with the presence of SA polymorphisms within these genomes; (iv) our Fst-MAF correlation is the first test (as noted by the reviewers) that allows researchers to distinguish between SA and sexually concordant selection, under the assumption that selection is relatively consistent across time. At minimum, we can therefore reject a model in which there are sex differences in the strength but not the direction of selection and selection is consistent over time (i.e., such a model is not a sufficient explanation for the patterns we report).**

**Overall, the above considerations are more clearly presented in the revision, issues concerning uncertainty in interpretation are now more plainly expressed, and readers should be able to come to their own conclusions.**

3. **Reviewer #3 was concerned that our analyses were insufficiently novel, though Reviewers #1 and #2 came to opposite conclusions regarding novelty. As noted by Reviewers #1 and #2: (i) our study reveals signals of SA selection in genomic data *while also controlling for population structure, possible technical artefacts and the high degree of sampling variance in Fst estimates,* which has not been done before; (ii) our study provides a simple framework for partitioning signals of SA polymorphism into viability, reproductive and overall fitness components (unlike previous studies which have focused entirely on viability), which is likely to find applicability in other systems.**

**Furthermore, our study develops a new test for distinguishing SA from sexually concordant selection (with caveats as mentioned above). Finally, our study allows us to examine the functional and evolutionary characteristics of candidate SA variants, for which there is little precedent. We have more clearly highlighted these novel characteristics of our study in the revised version.**

**Sincerely,**

**The authors**

**Reviewer #1**

This paper reports there are genome-wide signals of sexually antagonistic selection (SAS) that can be detected in the UK Biobank data. SAS is gaining traction as a research topic, and there has been recent controversy over whether the Biobank and other datasets show evidence of it. I like this paper for several reasons, but unfortunately there appears to be a fatal flaw so I cannot recommend publication.

The good news: aspects of this study are very well done. The statistical controls for population structure (which is a major concern) appear to be state-of-the-art. My favorite bit of the paper is the analysis of reproductive success: to my knowledge, other studies of SAS have not done that, and it provides a clever control for allele frequency difference in adults caused by things other than SAS (e.g. population structure). In general, the number and depth of the analyses is impressive.

We thank the reviewer for their positive overall assessment of our manuscript. Along with a number of important issues (which we respond to in turn below), the reviewer suggests that one particular aspect of our analysis—the absence of LD-pruning when assessing polygenic signals of SA selection—represents a “fatal flaw” in our analysis strategy. While we agree that the effects of LD-pruning on our results could have been analysed and discussed at greater length, LD alone cannot drive a signal of SA selection (for reasons that we expand on below). Given that the main conclusion of our paper remains unchanged with or without LD-pruning, we cannot agree that the absence of such an analysis in the original manuscript represents a fatal flaw.

Major comments

1. The big problem: it seems there is no control for the effects of linkage disequilibrium. A single SNP that is the target of SAS will generate the false appearance of SAS at other sites with which it is in LD. A standard way people use to deal with this problem is to thin the SNPs so that no more than one within a block of chromosome that has high LD. Of course this will usually throw out the sites that are actual targets of SAS. One could either hope that the selected sites would hitchhike enough to be detected, or perhaps use some clever approach to choose sites in a way that enriches for targets of SAS. There are probably yet other strategies, but something must be done about the effects of LD and hitchhiking.

In addressing this point, it is useful to distinguish between two separate questions our study addresses:

First, is there an overall, polygenic signal of SA selection within the human genome (e.g., elevated mean Fst, genome-wide; excess observations in the upper quantiles defined under our null models)?

Second, which SNPs represent the best candidate loci responding to SA selection?

Regarding the first question, LD alone is not sufficient to generate a polygenic signal of SA selection. However, we expect that any genome-wide signal of SA selection will involve hitchhiking of SNPs that are in LD with causal SA variants. This was not particularly clear in our original submission. We now clarify the point both verbally in the main text, and formally in the supplementary material (we have added a model showing how hitchhiking effects can contribute to excess divergence).

Beyond the fact that LD *per se* cannot explain the excess differentiation that we observe in the Biobank dataset, there are three further reasons why the polygenic signals of selection that we report are genuine and not artefactual:

1. Following the suggestion by Reviewer 1, we have repeated our analyses using a set of LD-pruned sites and this analysis shows that the same polygenic signals remain after this control (Figs. S3-4,S11-12).
2. In the original manuscript, we generated an empirical null via permutation of phenotypic values (i.e., permutation of sex labels in the case of adult and gametic Fst; permutation of LRS in the case of reproductive Fst) while maintaining the LD structure in our data (i.e. without LD-pruning). If the reviewer was correct and the signals of polygenic SA selection in our observed data had been artefactual, we would have observed an enrichment of high-Fst sites and elevated mean Fst in permuted data as well. Instead, the observed data show clear elevations relative to the permuted data. Moreover, the permuted distribution is very close to the theoretical null distribution, which validates the utility of the latter.
3. We presented functional analyses of candidate SA sites in our original manuscript that clearly revealed non-random (positive) associations between Fst and MAF, and between Fst and genic status. Both of these associations would not be anticipated if the signals had been artefactual—nor, indeed, do we see such associations systematically arise in the permuted data or simulations from the theoretical null.

The second question (i.e., which candidate SNPs respond to SA selection) is a more challenging issue to address. As we acknowledged in the original submission—but with added emphasis in the revision—the set of SNPs in the upper tail of the distribution of each Fst metric represent “candidates”, and these SNPs necessarily represent a combination of: (a) false positive SNPs that are completely unresponsive to any sex differences in selection and happen to, by chance, reside in the tail of an Fst distribution, and (b) SNPs that are responding to sex differences in selection, either because they are causal or because they are in LD with a causal SNP. This distinction does not undermine the downstream analyses that we have carried out on SNPs in the tails of Fst distributions.

Overall, we did not mean to imply that the polygenic signals we observed were *solely* driven by causal SA SNPs. Rather, the polygenic signals must be attributable to some fraction of causal SA polymorphisms and hitchhiking SNPs that are linked to causal SNPs. We have amended the language in our revised manuscript to make the distinction clearer (L141-149). We have also added an Appendix (SI Appendix C) that clarifies the expected degree of inflation in Fst for causal sites and linked sites.

2. Some of the results depend on a model with assumptions that I don’t think we can have much confidence in. The model laid out in SI section B assumes that all allele frequencies are at their deterministic equilibrium, and that relative fitnesses are constant in time. On the first assumption, I see no reason why some of the polymorphisms might not be transient. On the second assumption, I could imagine that some SAS results from some form of sexual selection, which typically is frequency-dependent and therefore violates the second assumption. Other kinds of temporal variation in fitness are also plausible.

In short, I would jettison the model and with it all the interpretations of the data that depend on it.

Our main purpose with this analysis was to outline a novel test for SA selection that can distinguish SA from SC selection. The reviewer is incorrect that our model in SI section B considers only deterministic equilibria; rather, it considers both deterministic equilibria and stationary distributions that incorporate both selection and drift (see, for example, the results that immediately follow the outline of our simpler deterministic model). We have clarified in the main text that our models do not rely on an assumption that the population is at deterministic equilibrium.

That said, the reviewer raises a good point that we assume relative fitnesses to be constant over time. If this is true, we expect the association between MAF and the magnitude of Fst to reflect the predominant form of sex-differential selection (i.e., SC versus SA). However, it is possible that contemporary patterns of selection (which influence between-sex Fst) are sufficiently different from historical patterns of selection (which influence MAF) that these models do not reflect the evolutionary causes of the observed association between MAF and Fst. In extreme cases, patterns of contemporary and historical selection could be completely independent, in which case, the intermediate-frequency polymorphic sites that are driving enrichment in Fst could be under some form of SC selection in the contemporary population, and are simply in process of sweeping through the population. Since we cannot definitively exclude this possibility, we indeed cannot claim that our test is an “unambiguous” consequence of SA selection. With this in mind, we have now included this alternative interpretation (L163-167, L336-340) and readers can evaluate whether (i) SA selection or (ii) temporally fluctuating and SC selection, represent a more parsimonious explanations for the positive correlation between Fst and MAF.

We have retained the test of association between Fst and MAF because it is novel (as pointed out by Reviewer #2) and it can potentially distinguish between SA and SC selection provided the fitness effects of polymorphisms are relatively consistent over time. Furthermore, inclusion of the formal theoretical predictions is certainly an improvement over verbal hypotheses for what we might expect to observe under specific scenarios of selection on each sex. Overall, it represents a valuable addition to this manuscript and to the field of sex differences, which currently lacks methods to detect SA selection in genomic data (Mank et al. 2017 *Nat Rev Genet*, Kasimatis et al. 2017 *J of Heredity*, Rowe et al. 2018 *Am Nat*, Ruzicka et al. 2020 *Evol Lett*).

3. Significance testing is done in two ways: by randomization, and using parametric tests. The two approaches seem to give consistent results, and randomization is more robust. I see no value added by the parametric tests. They can be omitted.

We agree that comparisons to empirical null distributions are more robust because they rely on fewer simplifying assumptions than the theoretical nulls. However, the theoretical null models have additional value because they explicitly show the factors that cause elevation in Fst in the absence of SA selection (unlike the empirical nulls), which is useful to researchers working with different datasets. For these reasons, we see value in retaining comparisons of observed values to both sets of nulls.

Minor comments

4. One of the criticisms raised by Kasimatis and others about large values of *F*ST reported in earlier studies is that the viability load is not plausible. While I don’t have an opinion on that, it’s an interesting question. The authors should figure out what load their results imply and say what they think about that.

We agree that this is an interesting question. However, it is very challenging to answer. Our results imply polygenic SA selection, but they do not tell us the number of causal loci or their distribution of effect sizes (as mentioned above, the overall elevation in Fst will be a combination of direct and hitchhiking responses to selection). This information would be required to make inferences about the viability load implied by our data. Moreover, our reproductive Fst metric requires no deaths, but instead reflects sex-specific variation for reproductive success, which is well-documented in humans (e.g., in the UK Biobank; Sanjak et al. 2017 *PNAS*) and is completely consistent with the inflation in reproductive Fst that we observe. An analysis of genetic loads is, therefore, not essential to the thrust of our arguments, though it is obviously something that future theoretical work might wish to explore.

5. Equations 1 – 4 can be moved to the SI: they are bookkeeping, and they give no biological insight. Equations 5 – 7 present the null distributions used in the parametric statistical tests, which in (3) above I suggested can be dropped. Absent all the equations, the ms would be more accessible to many readers.

While equations 1-4 do not give biological insight in themselves, it is difficult to follow the logic of our partitioning scheme, or our results, without presenting them. We now present equations 2-4 as equations 2a-c, and equations 5-7 as equations 3a-c, to make the presentation more accessible and to facilitate parallels between Fst metrics and their respective null distributions. If the editors feel that equations 1 and 2a-c intrusive in the main text, we can move them to the Methods or Supplementary Material.

As for the null distributions (now eqs. 3a-c), they are novel, simple, and essential to retain in the main manuscript text. They provide practical baselines for what to expect in relevant empirical datasets that may, in future, be used to evaluate between-sex Fst (viability or reproductive), and in this case are worth presenting because they speak directly to the effects of experimental design and sampling on the plausible distributions of between-Fst statistics.

6. Figures 2 – 4 seem to add very little and could be dropped. Figure 1 could be made visually stronger.

Figure 1 presents a schematic which is essential to understand our general approach (as also pointed out by reviewer #3), which we would be eager to strengthen, though it’s unclear what the reviewer is specifically suggesting should be changed. Figures 2-4 (now Figs 2-5) succinctly summarise our key empirical results and are therefore essential components of the manuscript. If we were to remove them, there would be no visual aid to summarise our results.

7. Line 97: Using genome-wide signals to detect SAS was a major point of Cheng and Kirkpatrick (2016).

We absolutely agree that Cheng and Kirkpatrick were the first to emphasize that, though there is little hope to detect individual SA loci (i.e., statistically significant single-locus hits) from Fst analyses, a polygenic or genome-wide signal of SA selection is potentially detectable. This is precisely the spirit in which we approach our analysis. We now cite this key paper in a revised version of the section that the reviewer refers to (L76).

We are also careful to clarify in the revised manuscript some of the limitations of previous analyses (including Cheng and Kirkpatrick’s) that have been highlighted in recent critiques, including its susceptibility to artefacts associated with population structure and mis-mapping of sequence reads, issues of statistical power, and limitations of previous work to effects of viability selection. As a result of these concerns, robust genomic evidence of sex-differential selection (let alone SA selection) is lacking in humans (L64-66, L312-316). These are the issues that we have sought to overcome in our manuscript.

8. Line 391 et seq.: Several previous studies reporting SAS using *F*ST between males and females have been disputed. The ms cites the papers by Kasimatis *et al*. but not Mank et al. (2020) Mol Ecol 29: 215 and the rebuttal to it.

We agree that these two papers should have been cited. We have now added both when discussing previous studies of SA selection using between-sex Fst.

**Reviewer #2**

This manuscript explores signals of sex-differential selection within human genomes. The extent of such selection, and particularly sexually antagonistic selection, has been the subject of some debate, with recent research and rebuttals reaching different conclusions.

This manuscript makes several advances in the area:

- Partitioning signals of sex differences in selection among fitness components (viability, reproductive success, total fitness)
- Removing loci that are potentially affected by alignment errors to the sex chromosomes
- Relating the evidence for sex-differential selection to the minor allele frequency (MAF)

The authors find evidence of a substantial number of loci with high Fst values, with 15-20% of SNPs falling in the top 1% of the theoretical null distribution (Figure 1). There was also a significant positive correlation between Fst values and MAF, which is consistent with the maintenance of polymorphism by balancing sexually antagonistic selection between males and females. By contrast, mutation-selection balance with sex-differential selection predicts that high Fst values would primarily be found among low MAF loci.

I think that this paper makes a major advance in a challenging and topical area.

We thank the reviewer for their positive assessment of our manuscript.

My main question had to do with the correlation with MAF. Even if there were many more loci subject to mutation-selection balance with sex-differential selection, wouldn't there be more power to detect Fst (higher chance of falling in the top 1% quantile) at those loci with intermediate allele frequency (i.e., balanced polymorphisms could be much rarer but have stronger signals)? More importantly, if there were no sites subject to balancing selection but some fraction of loci undergoing directional selection (with sex differences in selection), wouldn't those also be more likely to be detected if the MAF is high because of power issues? Changes in direction of selection due to modern medical care and modern human lifestyles could be leading to substantial numbers of weakly selected sites whose sign of selection has changed, with intermediate allele frequencies and slight sex differences in selection. Simulations would have to be conducted to test this alternative, but it seems plausible that the patterns are consistent with no balancing selection, just sex-differential selection with those sites under recent shifts in the direction of selection driving the Fst-MAF correlation.

Indeed, the observation that the high Fst loci aren't associated with particularly old alleles is more consistent with recent changes in the direction of selection on alleles in a sex-differential way.

The reviewer’s comment echoes the point raised by Reviewer #1—that a positive correlation between MAF and Fst could be driven by patterns of contemporary sex-differential selection that are not representative of patterns of selection in the past. We think that SA selection on genetic variants represents the simplest explanation for the positive association between MAF and Fst, as it is consistent with previous evidence for SA selection at the phenotypic level in this and other populations (Sanjak et al. 2017, *PNAS*; Stulp et al. 2012, *Biol. Lett.*; Stearns et al. 2012, *Proc. B*). Nevertheless, we agree that more complicated models of sex-differential selection, in which intermediate-frequency alleles with SC effects have undergone temporally fluctuating selection, could, in principle, drive the positive correlation between MAF and Fst. We have tempered our language and refer to the pattern as “consistent with SA selection”, rather than an “unambiguous” signal of SA selection. We also explicitly mention the assumptions of our model in the Theoretical Background section that appears early in the paper, and in our Discussion (L163-167, L336-340).

Line 35: "Contrasting" -> "Applying"? Doesn't seem like a contrast.

Given that our models are null models for what we expect to observe in the absence of sex-differential selection, we have amended this to “comparing”, which seems more apt than “applying”.

Line 49: First two sentences of Significance statement might be clearer as just one: "Differences between females and males represent a potent source of selection that
can, in principle, leave signals in genome sequence data."

The Significance statement has been removed in the new version of the manuscript.

Line 426 and 458: When first mentioning the tests on Hardy-Weinberg, refer to Sup Info Section C and mention that this would eliminated sites under substantial sex-differential selection, but that the test was set not to do so for weak to moderately strong selection. Perhaps, state that this test was needed to help eliminate mapping errors and leads to a slightly conservative test of sex-differential selection.

Amended (L439-441).

Line 584: Clearer as: "If candidate SA variants experience sufficiently strong balancing selection maintaining a polymorphic equilibrium,"?

Amended (L578).

Throughout: I couldn't figure out the number of permutations carried out.

We performed a single permutation of phenotypic values, which is justifiable given that we are not interested in establishing significance at a single-locus level but rather across many loci. Now clarified in the Results (L186-187), as well as the Methods (L514-524).

Figure S3 - I think the top three panels might be worth adding to the main text. These really jump out visually and emphasize just how different the Fst distribution is, particularly for reproductive success (that wasn't so obvious from the text).

This echoes a point made by Reviewer #3. We have added figures showing the bootstrapped distribution of observed minus null Fst (now panels D-F in Fig 2), and observed minus null correlation between Fst and MAF (now panels D-F in Fig 3), to emphasise the differences between observed and null data.

Figure S5 (or near there) - Could the allele frequencies in males be drawn versus the allele frequencies in females? This would allow us to see that the effect on allele frequency is small (lines 225-230), perhaps highlighting in color those that are in the top 1% Fst quantile and noting that none survive multiple corrections?

We have added a figure showing the allele frequency correlations in the Supplementary Information as Fig. S7. Because the allele frequencies are so similar, visually showing the largest allele frequency differences is not practicable. Nevertheless, the Manhattan plot (Fig. S6) makes the point that no SNPs survive Bonferroni multiple testing correction.

**Reviewer #3:**

This ms presents an analysis of human genome data in search of signals of sexually antagonistic selection. The Authors report evidence that the observed data is enriched for loci with high Fst values, relative to theoretical and empirical null expectations. In contrast, analyses aiming to use metrics for which balancing selection is expected to generate specific values did not detect support for balancing selection maintaining segregating variation. Below I raise some "major" questions about the significance of the work, and the analytical metrics and their interpretation, as well as some more minor questions and suggestions for clarification.

Overall, I struggled to understand how this work represented a significant advance in our understanding of the role of SA or how to detect it. Perhaps as a simple consequence of re-iteration of the same pattern, I didn't think that including LRS was particularly compelling, rather than a small addition to pre-existing inference of divergence in allele frequency between the sexes. I thought that the Discussion, in particular, needed to work harder to contextualise the current study. How do the results of this study fit with previous analyses of human genomic data? I feel a little overwhelmed by the number of studies that suggest the widespread presence of SA - so what is so novel about the current work?
• A specific breakdown of which previous papers do and do not support SA, and how the contradictory inferences can be interpreted would have helped me to understand better how to interpret this paper in relation to previous human studies.
o While the argument is made that it's hard to detect individually significant loci, is that really sufficient to explain why you are drawing a different conclusion to previous studies?
o Presumably there should be a correlation between the Fst estimates in your study and previous studies of the same data? Is it not possible to determine whether your approach and previous analyses are consistent?

In contrast to reviewers #1 and #2, both of whom are generally positive about the novelty of our analyses, reviewer #3 does not share this opinion. Below we clarify why we disagree. We have also made numerous changes in the manuscript aimed at better contextualising our work.

In response to the issue of novelty, there are several answers, which we now expand upon in a revised Introduction and Discussion:

1. Ours is the first study to present unambiguous signals of sex-differential selection in human genomes, particularly signals that survive controls for several potential artefacts that are now known to arise in studies of between-sex *F_ST_*. These issues include: uncontrolled population structure, mis-mapping of sequence reads between sex chromosomes and autosomes, and failure to properly account for the (often high) degree of error in estimates of between-sex *F_ST_*. Several previous studies have analysed human genomes with similar purpose to ours, including: (i) Lucotte et al. (2016, *GBE*); (ii) Cheng and Kirkpatrick (2016, *PLOS Genetics*); and (iii) Kasimatis et al. (2021, *Genetics*). However, all previous studies on SA polymorphism, with the exception of Kasimatis et al. (2021), have employed sample sizes that are at least are two orders of magnitude smaller than ours. Furthermore, no study to date (including Kasimatis et al. 2021) has tested for, or detected, a polygenic signal while also controlling for the mentioned artefacts.
2. As highlighted by reviewers #1 and #2, we present a framework for detecting SA polymorphisms affecting total fitness (rather than pre-adult survival) and for partitioning of signals into reproductive and non-reproductive components of total fitness. This stands in contrast to all previous research on the topic (in humans or any other taxa), which has focussed primarily on SA viability selection (e.g., Eyer et al. 2019 *PNAS*; Bissegger et al. 2019 *Mol Ecol*; Wright et al. 2018 *Mol Ecol*; Dutoit et al. 2018 *Mol Ecol*; Flanagan and Jones 2017 *Evol*; Chang and Kirkpatrick 2016 *PLOS Genet*) or SA selection on reproductive fitness components (Ruzicka et al. 2019, *PLOS Biol*).
3. We present a new test that is capable of distinguishing SA selection from SC selection—provided fitness effects of polymorphism are relatively consistent over time (see replies above). Given the paucity of tests to detect SA selection in genomic data in general (let alone tests for distinguishing SA from SC selection) this represents a significant advance on previous research.
4. We are able to examine the broader role of SA variants by examining their functional and evolutionary properties, which remains an open question. An examination of functional and evolutionary properties of genome-wide SA variants was presented Ruzicka et al. (2019, *PLOS Biol*) in *D. melanogaster*. However, other studies have assumed evolutionary mechanisms affecting SA variants *a priori*, which is not appropriate given that SA variants can exhibit a broad range of evolutionary histories (see Ruzicka et al. 2020, *Evol Lett*).

While I found much of the explanation of how to calculate and interpret the different Fst metrics, and the null, I nonetheless found the Results more challenging to follow. In particular, I felt that the specific metrics presented in the Results are based on the assumption that all of the analysed loci are sexually differentiated and sexually antagonistic, and that this is not appropriate (particularly, as this ms appears to focus on promoting an analytical approach for wide-spread application).

This is a good point, and highlights one of the issues in our presentation, which we have worked to correct (L141-167). It also echoes a point by Reviewer #1. Our null models are appropriate when there are no sex differences in fitness effects of polymorphism—a scenario for which we have very clear theoretical expectations, which aligns well with the empirical null (permuted data), and provides a baseline against which to test for elevations in Fst attributable to sex differences in selection.

We see such an elevation, which is consistent with polygenic sex-differential selection. As we now emphasize, the total elevation in Fst (the mean and the enrichment of polymorphic sites in tails defined under the null), will be due to sites that are directly responding to selection (causal sites) and sites that are hitchhiking with causal sites. We have added an Appendix (SI Appendix C) to formalize the effect of causal and hitchhiking polymorphisms on the inflation of Fst, and we make the point explicit (verbally) in the main manuscript.

Specifically,
• The biological logic of testing for a difference in mean differentiation between the observed and null Fst distributions was not clear to me. In contrast, I thought that the excess of loci in the upper quantile of the theoretical null Fst was an intuitively clear evidence of a signal of loci with divergence in allele frequency between the sexes. I thought that this was sufficient support for the interpretation of polygenic divergence bw the sexes, and did not get the importance of the mean Fst exceeding the null expectation (given that the mean is driven by some loci with large Fst).

Comparing mean Fst in observed and null data shows that the distribution of Fst for each metric is shifted towards higher values in observed relative to null data. Indeed, this test is more powerful than comparing loci in the bottom 99% and top 1%, since it assesses the cumulative signal across all loci, rather than partitioning the data in a (necessarily arbitrary) way. For example, one could imagine that very small elevations in the top 1% quantile would not be sufficient to show up as statistically significant, even when mean Fst is genuinely elevated when considering all loci cumulatively. Both tests are complementary. We have therefore retained them both and explained the logic behind the tests in more detail (L525-533).

o a question about the opposite pattern, and what is theoretically possible and biologically interpretable: if there are an excess of loci falling in the upper null quantile, then do you expect there to be a deficit (i.e., <1% of loci) concentrated in any particular quantile, or dispersed across the null distribution? Would a deficit of low Fst loci also be evidence of SA? Or, would a lack of divergence from the null expectation of proportion in the lowest null quantile be evidence against the presence of multiple loci under sexually congruent selection?

We agree that, as a corollary to the excess of high-Fst loci, a deficit of low-Fst loci is to be expected under polygenic SA selection. It is not clear why a lack of divergence from the null in the lowest null quantiles would be evidence against sexually concordant selection. It is important to note that our null model does not assume that all loci are neutral—rather, it assumes that there are no sex differences in selection. For example, if (though this is implausible) all loci were under selection of equal direction and magnitude in each sex, we would expect a distribution of Fst values that matches the null distribution. We have clarified this in the revised manuscript (L132-133).

• While the simulations (Appendix Section B) demonstrate that positive or negative slopes are generated for Fst against MAF under different evolutionary scenarios, to estimate the monotonic correlation across all observed loci again seems nonsensical in presuming then that all observed loci are evolving under the same mechanism (either balancing or stabilising selection). The observation of an excess of loci with extreme Fst (i.e., in the upper percentile of the null distribution) seems to define a set of candidates (which will contain false positives) that we can predict will have a higher MAF than other loci. Why is that hypothesis, based on presuming a different evolutionary process determining allele frequencies at different loci, not a more appropriate applied test of the prediction from the simulations that MAF and FST will jointly increase under SA selection?

The reviewer is correct that the Fst-MAF correlation test might be capturing loci that are evolving under a mixture of evolutionary mechanisms (e.g. balancing, directional selection, and drift). The value of this test is that loci in the upper quantiles of the observed Fst distribution are predicted to exhibit elevated MAF under one evolutionary mechanism (balancing selection or drift), whereas they are predicted to exhibit lower MAF under another mechanism (purifying selection). So, while the test cannot pinpoint the evolutionary mechanism affecting each individual locus, *it can rule out the possibility that sex differences in purifying selection alone* *are sufficient to explain UK Biobank patterns.* Some alternative mechanism must therefore be invoked. SA polymorphisms evolving under drift or balancing selection seems the simplest candidate, and would be consistent with previous reports of SA selection on traits such as height (Sanjak et al. 2017, *PNAS*).

Nevertheless, we did not mean to imply that our test demonstrates that *all* candidate SA sites are evolving under balancing selection or drift; rather, a sufficient number must be, or else we would not observe a positive correlation between Fst and MAF. We have clarified this in our revision (L168-169). We have also revised our manuscript to acknowledge that more complicated scenarios of selection (e.g., sex differences in selection + temporally fluctuating selection) could also, conceivably (though this remains to be shown) drive a positive correlation between Fst and MAF. Thus, our data is “consistent with” SA selection, as we state (L89, L335-340).

I did not feel thoroughly convinced that you were able to breakdown the data to consider signals of viability and reproductive fitness.
• Eqn 3 accounts for only a subset of individuals surviving to reproduce, but how does it account for loci with different effects on different fitness components and enable detection of loci with specific effects on each fitness component?
• What is the observed relationship between p & p', and what is the interpretation of loci that deviate from the norm of that relationship? As noted in the opening of the introduction, there may be antagonistic effects of loci on different fitness components, but it is not clear to me how this analysis accommodates e.g., alleles that increase male survival but decrease male LRS.

We agree with the reviewer that loci affecting viability and reproductive fitness components in similar manner, or loci affecting viability and reproductive fitness components in an opposing manner (antagonistically pleiotropic variants), could be interesting to investigate in their own right and that our framework for partitioning fitness components provides an avenue to do so. Since the focus of our manuscript is on sex differences in selection, our metrics were not designed to investigate such variants. Nevertheless, we plan analyses of antagonistically pleiotropic variants in future, so we have mentioned (L356-357) that developing metrics that are specifically suited to analysing antagonistic pleiotropy (within and between sexes) would be a fruitful topic for further research.

Minor comments and suggestions for clarification:
o Figure 2 and associated results:
♣ Line 211, 217, 222 refer to Fig. 2A,B,C as supporting the result of a difference between observed and nulls in the mean Fst, but as the mean Fst is not plotted I did not think this was helpful.

We agree that the figure does not illustrate differences in mean Fst well. Rather, it illustrates enrichment of loci in the upper quantiles of the null distribution. On this suggestion (and a similar suggestion by reviewer #2), we have added panels which show more clearly that mean Fst is much higher than expected by chance (new Fig. 2D-F).

♣ I couldn't figure out how Fig. S1 differed from Fig. 2, except the addition of the two other metrics. Is it all necessary (and if so, how do the top 3 panels differ between SI and main figure?).

The reviewer is correct that the top three panels of Fig S1 are the same as Fig. 2. We have now clarified that this is the case in the figure caption. The reason we have retained them is to allow readers to compare and contrast polygenic signals obtained using Fst and complementary statistics that control for population structure.

♣ Fig. S3 - How should we interpret the big difference between empirical and theoretical nulls for the reproductive Fst?

While we cannot pinpoint the exact reason why the empirical null differs marginally from the theoretical null for reproductive Fst, it is possible that some discrepancies arise due to simplifying assumptions used in obtaining Eq. 3b. For example, Eq. 3b assumes that genotype frequencies in the sample conform to Hardy-Weinberg equilibrium, yet this will not always be true. Such deviations could contribute to divergence between theoretical and empirical nulls. It is also worth remembering that the observed data shows significant elevations relative to both nulls, which is the more important point of comparison here.

♣ Figure 2 top panel: The relative pattern of the observed data is difficult to distinguish here - is it important to plot the individual points for the null distributions, or would it be possible to use e.g., shaded bands for the null while plotting the observed?

We have plotted individual points to avoid “summarised” versions of the data (e.g. shaded bands), and to allow readers to fairly compare observed and permuted data. (The only time we used summarised versions of the data is when plotting the associations between Fst and genic enrichment, which are weaker and thus benefit from the emphasis of a loess curve.) Nevertheless, we have narrowed the y axes in Fig. 2A-C to emphasise the difference between observed and null data.

♣ Figure 2 bottom panel: what you are plotting here is not dealt with in the Appendix (where Fst is plotted against MAF), so how do we interpret the plot of MAF against the rank with respect to the predictions?

While we agree that it would be ideal to visualise correlations between Fst and MAF at the level of individual loci, this cannot be done in a visually appealing way. We have therefore chosen to avoid this less aesthetically pleasing presentation in the main text (though it appears in the Appendix). The interpretation of the correlation between Fst and MAF remains unchanged.

If the reviewer is referring to the fact that we use a Spearman’s rank correlation to relate Fst and MAF, we note that the results are equivalent if using a Pearson’s correlation. We have chosen the former because neither Fst nor MAF distributions conform to normality assumptions, and the Spearman’s rank correlation is therefore more appropriate.

• Figure 1 - In general, I think it's great to have this figure to help us orient ourselves, but:
o I found the variable number of gametes (right panel) difficult to understand -what is the purpose of illustrating males with the same genotype producing different numbers of gametes?

We agree that this was not necessary. We have now changed the number of gametes for the male-beneficial homozygote genotype to be the same (revised Fig. 1).

o How do the two fitness components act independently? It seems that the increased transmission of one allele in males and the alternative in females is a simple consequence of their different frequency in the population, with no further/independent effect on reproductive success. Is there something additional that I should be understanding from this figure?

We were intending to illustrate that each respective allele has additional effects on reproductive success in each sex: for example, the frequency of the male-beneficial allele in the population is higher in male gametes (7/9) than it is in adult males (5/8), with the same applying for the female-beneficial allele in females. Nevertheless, we acknowledge that the contrast was not sufficiently sharp. To this end, we have further exaggerated the difference in allele frequency between adults and gametes in a revised Fig. 1.

o LRS is defined in the text (Line 137) after we are sent to Figure 1, so it would be helpful to have it written out in full in the legend for Figure 1.

Amended (L885).

• ~Line 145: I think it would be really helpful in here to be explicit about what is directly observed in the data versus what is inferred by calculation of something. I think that Mij / Fij are directly observed, but my understanding going forward would be better if you were explicit here about that.

This is correct, and now mentioned (L114-115).

• By restricting the data to post-reproductive I understand you get the estimate of LRS, but how does post-reproductive mortality before 45 impact inferences from adult Fst vs reproductive Fst?

Post-reproductive mortality before 45 should, if anything, accentuate signals of viability Fst, since those individuals will not be included in the sample of adults that form the basis of all analyses. Such mortality is likely to be minimal, given published age-specific mortality rates for the UK. In response to this and another comment, we have mentioned that further work aimed at disentangling the relationship between variant effects on viability and reproductive fitness components would be fruitful (L356-357).

• Line 105: I think it would be more appropriate to report up-front the number of individuals that you are able to include in your analysis, rather than the (~2x) number in the dataset - of course, even the reduced number of 250K is seriously impressive. You can clarify on Line 188 that you have a reduced sample due to exclusion of samples for data reasons. Can you also explicitly state somewhere which number (500K or 250K) was used to generate the null expectations for each Fst.

Amended (L85, L171).

• Line 367: Given the different approach that you appear to take to inferring widespread presence of SA (rather than seeking to support individually SA loci), then a demonstration of the same pattern in alternative datasets would be strong support for your conclusions.

We agree that this would be a valuable finding. However, to our knowledge, the UK Biobank is the only publicly accessible dataset that carries information on male and female LRS, which would be necessary to replicate the analyses presented here. For example, the Biobank VU used by Kasimatis et al. (2021, *Genetics*) only contains information on male and female allele frequencies, not their LRS.

• Line 378 vs 383 - occurring historically versus too recent to detect. Would be helpful to contextualise these apparently contradictory statements.

Amended (L364-377).

• Line 501: why that subset of SNPs to construct the relatedness matrix? What were the MAF of these?

It is standard practice to LD-prune SNPs before constructing a relatedness matrix (e.g. Speed et al. 2012, *Am J Hum Genet*), hence our decision. We used SNPs with MAF>0.01 in the LD-pruned data. Now clarified (L490).

• Line 528: while I was pleased to see, when reading the Results, both theoretical and empirical nulls, I did not find the set-up for the empirical null convincing. Why would one permutation of re-assignment of the entire genotype (and phenotype) between the sexes generate an informative comparison for the null expectation when the sexes are not under different selection?

We agree that a single permutation would have been inadequate as an empirical null if our focus had been on establishing statistical significance *at the single SNP level* (in which case, 1,000 or more permutations would have been preferable, though computationally challenging). However, our focus is on comparing the distribution of Fst values across many SNPs and performing a single permutation of phenotypic values allows us to obtain a null distribution of Fst values in the absence of sex differences in selection. This null distribution can be statistically compared, with adequate power, to the observed data, because the number of loci is large (i.e., sufficiently large that more permutations would yield statistical estimates that are similarly precise). Now clarified (L514-524).

If the substance of the criticism is about the way the permutations were done (as opposed to the number of permutations), the reviewer has not made clear why the permutation procedure is inappropriate.

• Line 540: Replicates of what?

Replicates of re-sampled SNPs. For example, one bootstrap replicate consists of a sample (with replacement) of N=544,635 SNPs, among which we calculated mean Fst in observed and permuted data. Now clarified (L525-533).

• Line 892: given that you don't seem to consider information on viability selection prior to reproductive maturity, but rather post reproductive senescence of females, then does this not simply apply at any point up to the age you define?

We consider information on viability selection prior to reproductive maturity—this is the basis for our tests comparing observed and null adult Fst. We agree that the statement could read just as accurately “In the absence of sex differences in viability selection”. We have amended this statement accordingly (L950).

• Line 908;915: It isn't clear to me from this wording if the adult or the offspring "carry the genotype". Consider re-arranging these sentences for clarity.

Clarified (L965-973).

• Line 1003: Can you clarify what you mean by this assumption of equal frequency between the sexes for loci passing quality control, and how this relates to the overall analysis to detect differences in allele frequency.

What we mean is that our data is quality-filtered such that very large differences in allele frequencies between sexes cannot occur (SI Appendix D); under these conditions, the null for reproductive Fst (i.e. eq. 3b), which we present in the main text, is accurate. Now clarified (L1060).

• Line 1029: you define modest to weak selection as s=0.2, and that the following is a reasonable approximation of sex-specific allele frequencies. On line 1068 we get to an approximation that is (extremely) accurate when s<0.1. How do these reconcile, and how do they relate to empirical estimates of s?

This was a typo. The first equation should have read s=0.1. Amended (L1086).

• Line 1087: what are the conditions for balancing selection / the criteria that the loci met?

The criteria for balancing selection (assuming co-dominance) are:

$$\frac{s_{f}}{1+s_{f}}<s_{m}<\frac{s_{f}}{1-s_{f}}$$

Clarified (L1142-1143).

• Line 1118: "Parameters include..." please clarify what distinguishes the panels. Further, how this value of s related to previously assumed values of s.

Clarified (L1166-1176). We have chosen a small value of s_max_ to reflect the weak-selection assumptions made throughout the manuscript.
